# Are There Socio‐Demographic Inequalities in the Utilisation of Tumour and ctDNA Somatic Mutation Testing in Solid Tumours? A Systematic Review

**DOI:** 10.1002/cam4.71668

**Published:** 2026-03-13

**Authors:** Sarah Rae, Annie Baldwin, Maria Julia Lagonera, Ruth Norris, Alastair Greystoke, Linda Sharp

**Affiliations:** ^1^ Newcastle University Newcastle upon Tyne UK; ^2^ Sir Bobby Robson Cancer Trials Research Centre, Northern Centre for Cancer Care, Freeman Hospital Newcastle upon Tyne UK; ^3^ Population Health Sciences Institute Newcastle University, Newcastle University Newcastle upon Tyne UK

**Keywords:** ctDNA, inequalities, next‐generation sequencing, oncology, Oncotype DX, socio‐demographics

## Abstract

**Introduction:**

Somatic mutation testing in solid tumours represents a rapidly advancing field which increases opportunities for access to molecularly targeted therapeutics and clinical trials. This systematic review determined whether socio‐demographic inequalities affect utilisation of novel somatic mutation testing.

**Methods:**

Following PRISMA 2020 guidance, MEDLINE, EMBASE, Scopus, CINAHL, Web of Science, PubMed and PsycINFO were searched for peer‐reviewed studies (January 2018–March 2025). Data was extracted reporting utilisation of novel somatic mutation testing panels, including Oncotype DX, for solid tumours by socio‐demographic measures. A modified International Society for Pharmacoeconomics and Outcomes Research (ISPOR) checklist assessed study quality. Unadjusted odds ratios (ORs) and 95% confidence intervals (CIs) were calculated where needed and narrative synthesis undertaken. Data was stratified by receipt of Oncotype DX testing and next‐generation sequencing (NGS) panels.

**Results:**

The 27,749 citations screened identified 24 studies meeting the inclusion criteria. These reported on two modalities of testing (Oncotype DX and other NGS sequencing panels) across five cancers. Twenty‐three studies were from US populations. These highlighted disparities in utility of testing across socio‐demographic measures and particularly decreased utilisation with increased age, non‐white ethnicity, lower socio‐economic status, and non‐private insurance. The mean study quality score by a modified ISPOR checklist was 8.3/10.

**Conclusion:**

These results provide a contemporary update on evidence of disparities in access to novel genomic testing. As an expanding field, this requires further investigation to prevent accentuations in inequitable implementation of precision oncology and differences in outcomes between different socio‐demographic groups.

## Graphical abstract

This systematic review investigates whether inequalities exist in the utilisation of tumour and ctDNA somatic mutation testing in solid tumours. The review concludes that, across tumour types, testing modalities and socio‐demographic factors, inequalities exist and require further exploration.

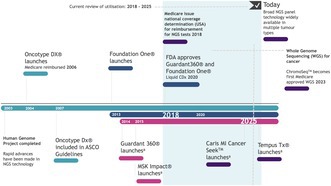

## 1 Introduction

It is recognised that inequalities in access to diagnostic and therapeutic modalities due to socio‐demographic status affect patients with cancer [1]. Patients from deprived backgrounds are less likely to have definitive surgical management of malignancy and adjuvant chemotherapy or radiotherapy; less likely to receive biological or precision therapies or immunotherapy; more likely to face substantial financial toxicity in attending appointments; and ultimately more likely to die of their disease [2, 3, 4, 5]. Similar patterns in equality of access to existing treatments are well characterised for older patients and patients from different ethnic backgrounds, such as disparities between women's outcomes in breast cancer between ethnic groups in New Zealand [6, 7, 8].

**FIGURE 1:**
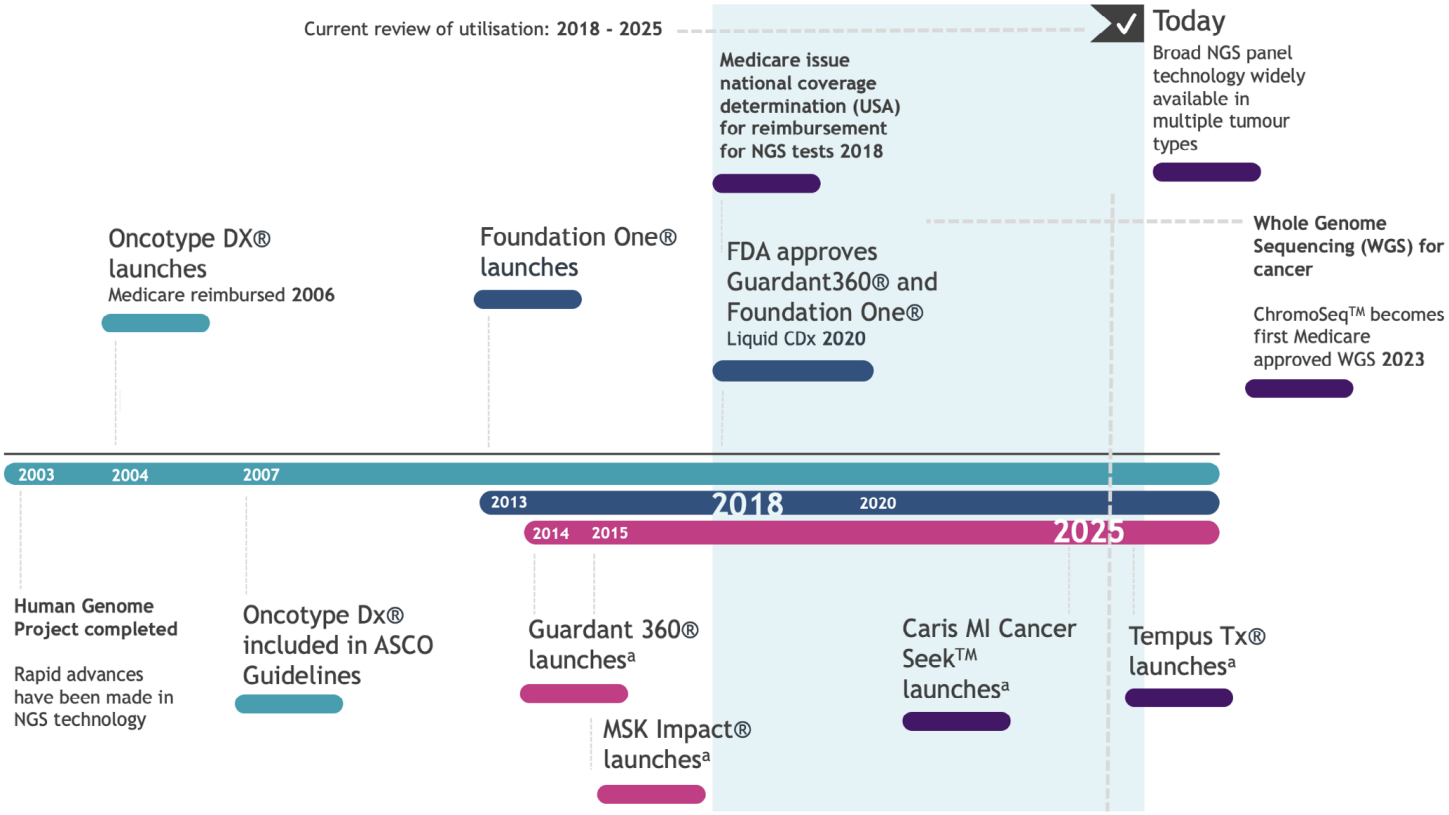
Timeline of advances in somatic mutation profiling in solid tumours [10, 11, 18, 19, 20, 21, 22, 23, 24, 25, 26]. Timeline of pertinent developments in availability of profiling for somatic mutations in solid tumours. Foundation One, Guardant 360, MSK Impact, Caris Oncology and Tempus Oncology currently represent examples of commercial panels within this field. Medicare reimbursement dates represent broadly increased availability of testing in the US. This demonstrates a significant shift in 2018 in the broadness of access to NGS technology for patients with cancer. Next line advances in WGS for solid tumours are highlighted in the rapidly expanding area of precision oncology. ^a^FDA‐approved products.

The previous decade has demonstrated rapid expansion of availability of commercially available next generation sequencing (NGS) based comprehensive genomic profiling technology for somatic mutations in tumours [9]. A timeline of major transatlantic milestones within this field is shown in Figure 1. Somatic mutation testing has been developed across both solid tumour testing and cell free or circulating tumour DNA (ctDNA). The first such ctDNA panel with Food and Drug Administration (FDA) approval was the Guardant360 Companion Diagnostic (CDx) assay in August 2020, followed within weeks by Foundation One Liquid CDx—representing how recently commercial availability of this testing has been realised [10, 11]. NGS testing allows for targeting of cancer therapeutics and generates information for prognostication and enrolment into clinical trials. Testing, therefore, advances our ability to realise the potential of ‘precision oncology’ from within the clinic. Some forms of panel sequencing tests have been available for longer—since 2004 in the case of the 21‐gene panel recurrence test score in breast cancer, also known as Oncotype DX. This is a predictive genetic test which does not utilise NGS but reverse transcription polymerase chain reaction (RT‐PCR) of 21 recognised genes involved in breast cancer to stratify patients into those who are at highest risk of recurrence and are therefore most likely to benefit from adjuvant chemotherapy. In 2013, Oncotype DX additionally launched a 17 gene panel for use in prostate cancer [12]. Our understanding of the utilisation of Oncotype DX panels, as such data reaches maturity, is only contemporarily being investigated [13, 14].

Socio‐demographic disparities in the access to and utilisation of broad panel somatic tumour mutation testing remain largely unexplored. Novel modalities are associated with high purchase costs, which may be presumed to be barriers to reaching the most disadvantaged and marginalised patients with cancer, especially in non‐publicly funded healthcare settings, although many varied healthcare systems support and reimburse costs for such testing [15]. However, in more highly tailoring initial therapeutics, utilising molecular data, this may provide a pathway for reducing current treatment inequalities [16]. Previously, Norris et al. explored socio‐economic inequalities in utilisation of predictive biomarker tests and biological and precision therapies for cancer and found there to be socio‐economic inequalities in predictive biomarker tests and biological and precision therapy utilisation [17]; that review included studies published up to 2019, although many included patients diagnosed years previously.

This current systematic review collates new literature, specific to tumour and ctDNA somatic mutation testing of solid tumours in relation to broader socio‐demographic factors since 2018, as an update to our understanding of the rapidly evolving literature in this field.

## 2 Methods

This review was registered with PROSPERO, the international database of prospectively registered systematic reviews (CRD42023487689). It is reported in accordance with the Preferred Reporting Items for Systematic Reviews and Meta‐Analyses (PRISMA) 2020 statement (Table S1).

### 2.1 Search Strategy and Study Selection

Seven databases (MEDLINE, EMBASE, Scopus, CINAHL, Web of Science, PubMed and PsycINFO) were searched. Articles published between January 2018 and March 2025 were included. This time period was selected to reflect the period during which multiple comprehensive genomic sequencing panels received approvals and reimbursement worldwide.

Both tumour sample and ctDNA somatic mutation testing were reviewed across all solid tumours. Germline testing and testing done in non‐malignant tumours were excluded. Studies exploring single gene tests in isolation were not included as these represented an older technology licensed significantly prior to 2018. Search terms covering socio‐demographic status, disparities in utilisation of, and comprehensive genomic sequencing panels were developed. Full search strategies are included in Supplementary Methods (Table S2).

The inclusion criteria for full‐text studies written in English were determined by a defined PICOS question as follows:

#### 2.1.1 Population

Solid malignant tumour diagnosis in a patient of any age or sex.

#### 2.1.2 Intervention

Utilisation of a comprehensive genomic sequencing panel for somatic mutation testing during malignant tumour management.

#### 2.1.3 Comparison

It was a requirement that a comparator was reported—where reported, this included—a clinical alternative or no comprehensive genomic sequencing panel being used.

#### 2.1.4 Outcome

Utilisation data reported by a socio‐demographic measure (e.g., race/ethnicity, median household income, age, education level, sex/gender).

#### 2.1.5 Setting

Retrospective or prospective observational study (including randomised controlled trials analysed as observational cohorts). A decision tree to determine eligibility of full texts is included in Figure S3.

Title and abstract screening was completed by one author (S.R.), with 10%+ confirmed between four authors (R.N., A.G., L.S. and A.B.). Disagreements were discussed and agreed with a senior author (A.G.). All full text studies were reviewed by one author (S.R.) and independently reviewed by at least one other author (R.N., A.G., A.B. or M.J.L.). Disagreements were discussed and if required resolved with the involvement of a third author (A.G.). Agreement between reviewers was excellent (*κ* = 0.96). Forward and backward citation searching was conducted for eligible studies as well as citation searching of previous reviews on related topics.

### 2.2 Data Extraction and Quality Assessment

Data was extracted by one author (S.R.) and confirmed by a second author (A.B. or M.J.L.). Disagreements were resolved through discussion with a third author (A.G.). Where data was unclear, the study authors were contacted by email. Studies were included only if responses met the inclusion criteria. Duplicate usage of the same database for overlapping studies was noted and considered in discussion.

Data extracted included: author(s); publication year; country; data source; number in study population; malignant solid tumour diagnosis; patient age(s); socio‐demographic measure(s) and unit (including measures of socio‐economic group); comparator(s) were included; and measures of association for not having testing completed using a comprehensive genomic sequencing panel by socio‐demographic status (e.g., OR, 95% CIs, *p*‐values, etc.). If adjusted and unadjusted analyses were reported, the adjusted results were extracted; if more than one multivariable analysis was conducted, information was extracted from the most comprehensive adjusted model.

All studies deemed eligible were quality appraised and scored using a modified version of the ISPOR checklist for retrospective database studies (Table S4) (modifications as per Norris et al.). Appraisal was completed by one reviewer (S.R.) and discussed with the review team (A.B., M.J.L., R.N., L.S. and A.G.).

### 2.3 Synthesis of Evidence

Data was synthesised using a summary of findings table. Where not reported (and where possible), percentages of utilisation of comprehensive genomic sequencing panels by socio‐demographic metric(s) were calculated from data reported in the included studies. Studies were noted to be heterogeneous in terms of outcome analyses, socio‐demographic comparisons made, whether ORs (crude or adjusted) were reported, and the variables that any adjusted ORs were controlled for. Unadjusted ORs were computed by the review authors when not reported to enable inclusion of as many studies as possible in the synthesis in a consistent way. The narrative synthesis divided by Oncotype DX testing and NGS testing (both tumour sample and ctDNA analysis) was completed for included studies. This division between Oncotype DX and NGS testing was completed due to recognition of variation in usage of testing modalities and differences in timeframe in which they were introduced to cancer care. Meta‐analyses of results were not conducted due to the small number of studies identified, and heterogeneity in socio‐demographic metrics and tests. Duplication of data sources further reduced those eligible for statistical combination.

## 3 Results

### 3.1 Search Results

**FIGURE 2:**
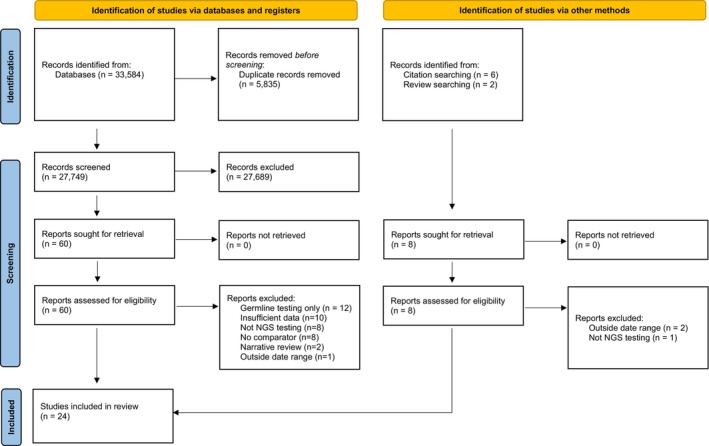
Study selection according to the PRISMA (2020) statement. NGS: Next Generation Sequencing [50].

The search identified 33,584 citations. There were 5835 duplicates removed and title and abstract screening of 27,749 records was completed to assess for eligibility. Of these, 60 studies were retrieved for full‐text review. Eight additional studies were identified by forward and backwards citation searching and citation searching of previous related reviews. Overall, 24 studies identified met the inclusion criteria, had appropriate comparators, and were included in the review (Figure 2, Table 1).

**TABLE 1:** Characteristics of included studies.

Oncotype DX test: breast cancer and prostate cancer															
Study	Sampling frame														
	Country	Data source	Study population^a^	Sequencing method overall utilisation (number, %)	Comparator	Measure	Utilisation (number [%])							*p*	QA
Acuna et al. (2021) [27]	USA, New Jersey	New Jersey Cancer Registry	Breast Cancer in Latina/Hispanic patients, New Jersey *n* = 5777 (1916 eligible for testing)	Oncotype DX *n* = 1167 (60.9)	No testing	Age (years)	< 50	50–59	60–69	70–79	> 80			< 0.001	9.5
							360/574	343/525	299/463	156/307	9/47				
							(62.7)	(65.3)	(64.6)	(50.8)	(19.1)				
							OR: Ref	OR: 0.97 (0.73–1.28)	OR: 0.89 (0.67–1.19)	OR: 0.43 (0.31–0.59)	OR: 0.08 (0.04–0.18)				
						Race^b^	White	Black	Other	Missing/unknown				0.829	
							1046/1727	53/85	62/98	6/6					
							(60.6)	(62.4)	(63.3)	(100)					
						Ethnic Subgroup	Spanish/Hispanic/Latino NOS	South/Central America	Other	Puerto Rican	Cuban	Dominican	Mexican	0.284	
							361/573	223/383	221/349	192/336	67/99	59/100	44/76		
							(63.0)	(58.2)	(63.3)	(57.1)	(67.7)	(59.0)	(57.9)		
							OR: Ref	OR: 0.93 (0.6–1.26)	OR: 0.87 (0.63–1.19)	OR: 0.76 (0.55–1.04)	OR: 1.6 (0.95–2.7)	OR: 0.97 (0.6–1.57)	OR: 0.82 (0.47–1.44)		
						Insurance	Insured	Medicaid	Uninsured	Insured, but not specified	Missing/unknown			0.018	
							740/1180	178/285	77/154	140/238	32/59				
							(62.7)	(62.4)	(50.0)	(58.8)	(54.2)				
							OR: Ref	OR: 1.32 (0.97–1.81)	OR: 0.58 (0.39–0.86)	OR: 0.92 (0.67–1.27)					
						Area based SES status^c^	Low	Low‐middle	Middle	Middle‐high	High	Unknown/missing^b^		0.001	
							196/365	219/359	198/323	239/399	299/448	16/22			
							(53.7)	(61.0)	(61.3)	(59.9)	(66.7)	(72.7)			
							OR: 0.58 (0.42–0.82)	OR: 0.82 (0.59–1.14)	OR: 0.77 (0.55–1.08)	OR: 0.76 (0.56–1.05)	OR: Ref				
Chen et al. (2022)^d^ [14]	USA, Pennsylvania	National Cancer Database	Breast cancer *n* = 429,658	Oncotype DX *n* = 186,505 (43.4)	No testing	Age (years)	Mean received testing: 58.9	Mean did not receive testing: 65.7			OR age at diagnosis as per 1 year increase: 0.944 (0.943–0.945)			< 0.0001	9
							(10.46)	(11.87)							
						Race	White	Black	Others					< 0.0001	
							162,232/374,267	13,875/32,306	10,398/23,085						
							(43.3)	(42.9)	(45.0)						
							OR: Ref	OR: 0.98 (0.96–1.01)	OR: 1.07 (1.04–1.10)						
								*p* = 0.17	*p* < 0.0001						
						Spanish/Hispanic origin	Hispanic	Non‐Hispanic	Unknown					< 0.0001	
							8204/19,830	173,086/397,482	5215/12,346						
							(41.4)	(43.5)	(42.2)						
							OR: 0.91 (0.89–0.94)	OR: Ref	OR: 0.95 (0.91–0.98)						
							*p* < 0.0001		*p* = 0.004						
						Insurance	Not insured	Medicaid	Medicare	Other Gov.	Status unknown	Private insurance		< 0.0001	
							2222/4973	9270/19405	57,009/82,108	2012/4375	1910/4456	114,082/214,341			
							(44.7)	(47.8)	(31.3)	(46.0)	(42.9)	(53.2)			
							OR: 0.71 (0.67–0.75)	OR: 0.80 (0.78–0.83)	OR: 0.40 (0.40–0.41)	OR: 0.75 (0.70–0.79)	OR: 0.66 (0.62–0.70)	OR: Ref			
							*p* < 0.0001	*p* < 0.0001	*p* < 0.0001	*p* < 0.0001	*p* < 0.0001				
						Median income quartiles (2013–2016)	< $40,227	$40,227–$50,353	$50,354–$63,332	> $63,332	Missing			< 0.0001	
							20,167/51,363	30,007/73,758	37,054/87,011	73,893/161,556	25,384/55,970				
							(39.3)	(40.7)	(42.6)	(45.7)	(45.4)				
							OR: 0.77 (0.75–0.78)	OR: 0.81 (0.80–0.83)	OR: 0.88 (0.87–0.89)	OR: Ref	OR: 0.98 (0.97–1.00)				
							*p* < 0.0001	*p* < 0.0001	*p* < 0.0001		*p* = 0.11				
						Location: metro/urban/rural	Metro	Urban	Rural	Unknown				< 0.0001	
							158,513/365,148	20,761/48,710	2558/6137	4673/9663					
							(43.4)	(42.6)	(41.7)	(48.4)					
							OR: Ref	OR: 0.97 (0.95–0.99)	OR: 0.93 (0.89–0.98)	OR: 1.22 (1.17–1.27)					
								*p* = 0.001	*p* = 0.0067	*p* < 0.0001					
Dunn et al. (2024) [28]^d^	USA, North Carolina	The Carolina Breast Cancer Study Cohort	Breast cancer *n* = 2998	Oncotype DX *n* = 609 (20.3)	No testing	Race	Non‐Black	Black							7
							401/938	208/677							
							(42.8)	(30.7)							
							OR: Ref	OR: 0.59 (0.48–0.73)							
								*p* < 0.0001							
Hull et al. (2018) [29]	USA, Massachusetts	VA Central Cancer Registry	Breast cancer in veterans (US Army) *n* = 328	Oncotype DX *n* = 82 (25.0)	No testing	Age (years)	20–49	50–59	> 59						8
							23/54	32/105	27/169						
							(42.6)	(30.5)	(15.9)						
							OR: Ref	OR: 0.59 (0.3–1.17)	OR: 0.26 (0.13–0.51)						
								*p* = 0.13	*p* < 0.001						
						Race	White	Non‐White							
							56/237	26/91							
							(23.6)	(28.6)							
							OR: Ref	OR: 1.29 (0.75–2.23)							
								*p* = 0.36							
						Sex	Female	Male							
							72/267	10/61							
							(27.0)	(16.4)							
							OR: Ref	OR: 0.53 (0.26–1.10)							
								*p* = 0.09							
						Distance from National Cancer Institute	≤ 60 miles	> 60 miles							
							64/223	18/105							
							(28.7)	(17.1)							
							OR: Ref	OR: 0.5 (0.28–0.9)							
								*p* = 0.02							
						Fee based care	Yes	No							
							26/138	56/190							
							(18.8)	(29.5)							
							OR: 0.56 (0.33–0.94)	OR: Ref							
							*p* = 0.03								
Iles et al. (2022) [30]	USA	National Cancer Database	HR+/HER2−, early‐stage breast cancer *n* = 530,125	Oncotype DX *n* = 255,971 (48.3)	No testing	Age group (years)	< 40	40‐69	70+						7.5
							9409/16,932	201,869/356,082	44,693/157,111						
							(55.6)	(56.7)	(28.4)						
							OR: Ref	OR: 1.01 (0.99–1.02)	OR: 0.57 (0.56–0.58)						
						Race/ethnicity	Non‐Hispanic White	Non‐Hispanic Black	Hispanic	Non‐Hispanic other					
							207,373/428,355	19,198/41,824	11,073/23,321	10,622/20,732					
							(48.4)	(45.9)	(47.5)	(51.2)					
							OR: Ref	OR: 0.94 (0.93‐0.95)	OR: 0.94 (0.93‐0.96)	OR: 0.99 (0.98–1.00)					
						Primary insurance	Private insurance	Medicare/Medicaid	Uninsured						
							152,357/266,378	100,561/254,767	3053/6353						
							(57.2)	(39.5)	(48.1)						
							OR: Ref	OR: 0.89 (0.88–0.90)	OR: 0.87 (0.84–0.89)						
Mukand et al. (2024) [31]	USA, Multiple States	National Cancer Institutes' Surveillance, Epidemiology and End Results (SEER) population‐based Cancer Registry Program	Localised Prostate Cancer Stage T1c–T2c *n* = 111,434	Oncotype DX Genomic Prostate Score *n* = 6014 (5.4)	No testing	Age group (years)	< 55	55–64	65–74	75+					9.5
							689/13,403	2414/44,515	2477/44,535	434/8981					
							(5.14)	(5.42)	(5.56)	(4.83)					
							OR: Ref	OR: 1.01 (0.93–1.10)	OR: 1.07 (0.98–1.16)	OR: 0.98 (0.86–1.10)					
						Race and ethnicity	American Indian or Alaska Native	Asian	Black	Hispanic	Native Hawaiian or other Pacific Islander	Unknown	White		
							< 15/276	218/4140	580/16,894	394/10,019	< 15/290	89/1263	4709/78,552		
							(< 5.43)	(5.27)	(3.43)	(3.93)	(< 5.17)	(7.05)	(5.99)		
							OR: 1.02 (0.59–1.76)	OR: 0.91 (0.79–1.06)	OR: 0.70 (0.64–0.76)	OR: 0.70 (0.62–0.78)	OR: 0.78 (0.42–1.45)	1.06 (0.85–1.32)	OR: Ref		
						Neighbourhood SES quintile (census tract)	Q1—Low	Q2	Q3	Q4	Q5—High	Missing			
							405/12,700	511/14,113	792/18,046	1,227/24,262	2550/35,293	529/7,020			
							(3.19)	(3.62)	(4.39)	(5.06)	(7.23)	(7.54)			
							OR: Ref	OR: 0.97 (0.85–1.12)	OR: 1.09 (0.95–1.24)	OR: 1.17 (1.03–1.33)	OR: 1.62 (1.44–1.83)	OR: 1.25 (0.94–1.65)			
						Insurance status	Insured	Any Medicaid	Uninsured	Unknown					
							5197/96,879	263/6287	21/904	533/7364					
							(5.36)	(4.18)	(2.32)	(7.24)					
							OR: Ref	OR: 0.93 (0.82–1.06)	OR: 0.56 (0.36–0.87)	OR: 1.26 (1.13–1.40)					
						Marital status	Married	Unmarried	Unknown						
							4239/77,508	1207/24,403	568/9523						
							(5.47)	(4.95)	(5.96)						
							OR: Ref	OR: 1.01 (0.95–1.08)	OR: 1.07 (0.97–1.19)						
Natsuhara et al. (2019)^d^ [32]	USA, Boston	Dana‐Farber/Brigham and Women's Cancer Centre Database	HR+ and HER2− breast cancer *n* = 498	Oncotype DX *n* = 309 (62.0)	No testing	Age (years)	< 50	50–65	> 6					< 0.0001	9
							85/120	170/247	54/131						
							(70.8)	(68.8)	(41.2)						
							OR: Ref	OR: 0.91 (0.56‐1.46)	OR: 0.29 (0.17‐0.49)						
								*p* = 0.70	*p* < 0.0001						
						Race	White	Non‐White						0.03	
							271/423	38/75							
							(64.1)	(35.9)							
							OR: Ref	OR: 0.58 (0.35–0.94)							
								*p* = 0.029							
						Insurance	Private	Public						< 0.0001	
							259/357	50/141							
							(72.6)	(35.5)							
							OR: Ref	OR: 0.21 (0.14‐0.32)							
								*p* < 0.0001							
Roberts et al. (2019)^e^ [33]	USA	Genomic Health's Clinical Laboratory Database	Breast cancer *n* = 30,401	Oncotype DX *n* = 4271 (13.9)	No testing	Age (years)	< 45	45–54	55–64	65–74		≥ 75			7
							9.1%/4271	24.8%/4271	31.1%/4271	25.1%/4271		9.9%/4271			
							OR: Ref	OR: 1.47 (1.3–1.68)	OR: 1.98 (1.75–2.25)	OR: 2.25 (1.97–2.57)		OR: 1.33 (1.13–1.56)			
						Race	Black	White	Other	Unknown					
							9.5%/4271	82.3%/4271	7.8%/4271	0.5%/4271					
							OR: Ref	OR: 1.12 (0.99–1.27)	OR: 0.93 (0.78–1.12)	OR: 0.86 (0.52–1.43)					
						Insurance	Insured	Nonspecific	Medicaid	Uninsured		Unknown			
							74.2%/4271	12.9%/4271	9.5%/4271	1.9%/4271		1.5%/4271			
							OR: Ref	OR: 0.99 (0.89–1.1)	OR: 0.86 (0.77–0.98)	OR: 1.05 (0.82–1.36)		OR: 1.16 (0.87–1.56)			
						Socio‐economic status (quintile)	1	2	3	4		5	Unknown		
							12.1%/4271	16.3%/4271	18.9%/4271	22.8%/4271		28.6%/4271	1.4%/4271		
							OR: Ref	OR: 1.2 (1.05–1.37)	OR: 1.25 (1.1–1.42)	OR: 1.36 (1.2–1.55)		OR: 1.6 (1.4–1.82)	OR: 1.37 (1.0–1.88)		
						Marital status	Married	Divorced/separated	Single/unmarried	Widowed		Unknown			
							59.2%/4271	12.2%/4271	14.5%/4271	9.8%/4271		4.2%/4271			
							OR: Ref	OR: 1.06 (0.95–1.19)	OR: 1.05 (0.95–1.17)	OR: 0.79 (0.69–0.89)		OR: 0.77 (0.65–0.92)			
Ko et al. (2020) [34]	USA	National Cancer Database	Breast Cancer, Stage I–II ER+ *n* = 387,008	Oncotype DX, Mamma Print, or other multigene signature test completed *n* = 147,863 (38.2)	No testing	Age (years)	40–50	50–70	≥ 70						9.5
							27,280/59,116	95,105/209,823	25,478/118,069						
							(46.1)	(45.3)	(21.6)						
							OR: Ref	OR: 0.95 (0.93–0.97)	OR: 0.33 (0.32–0.34)						
								*p* < 0.0001	*p* < 0.0001						
						Race	White	Black	Other						
							130,081/336,982	11,740/34,173	6042/15,853						
							(38.6)	(34.4)	(38.1)						
							OR: Ref	OR: 0.82 (0.8–0.85)	OR: 0.87 (0.84–0.9)						
								*p* < 0.0001	*p* < 0.0001						
						Hispanic Ethnicity	Non‐Hispanics	Hispanics							
							141,879/369,309	5,984/17,699							
							(38.4)	(33.8)							
							OR: Ref	OR: 0.78 (0.75–0.81)							
								*p* < 0.0001							
						Insurance	Private	Medicaid/other gov.	Medicare	Uninsured					
							89,869/195,825	8963/23,397	46,976/161,886	2055/5900					
							(45.9)	(38.3)	(29.0)	(34.8)					
							OR: Ref	OR: 0.80 (0.78*–*0.83)	OR: 0.82 (0.8–0.83)	OR: 0.70 (0.66–0.74)					
								*p* < 0.0001	*p* < 0.0001	*p* < 0.0001					
						Median income	< $30,000	$30,000–$35,999	$36,000–$45,999	$46,000+					
							12,617/36,349	21,051/58,184	38,590/102,585	75,605/189,890					
							(34.7)	(36.2)	(37.6)	(39.8)					
							OR: 1.04 (1.01–1.08)	OR: 1.02 (1.0–1.05)	OR: 1.03 (1.01–1.05)	OR: Ref					
							*p* = 0.01	*p* = 0.111	*p* = 0.005						
						Education status—% with no HS degree	< 14%	14%–19.9%	20%–28.9%	≥ 29%					
							68,740/171,392	34,469/91,249	28,473/76,574	16,181/47,793					
							(40.1)	(37.8)	(37.2)	(33.9)					
							OR: Ref	OR: 0.94 (0.93–0.96)	OR: 0.94 (0.92–0.96)	OR: 1.04 (1.01–1.08)					
								*p* < 0.0001	*p* < 0.0001	*p* < 0.0001					
Van Alsten et al. (2024) [35]^d^, ^i^	USA	The Carolina Breast Cancer Study Cohort	Breast Cancer, Stage I–II, HR+/HER2− *n* = 1615	Oncotype DX *n* = 609 (37.7)	No testing	Age	< 50	> 50							7
							325/869	284/746							
							(37.4)	(38.1)							
							OR: Ref	OR: 1.03 (0.84–1.26)							
								*p* = 0.78							
						Income	< 50k	30–50k	15–30k	> 15k					
							319/739	116/301	99/272	52/215					
							(43.2)	(38.5)	(36.4)	(24.2)					
							OR: Ref	OR: 0.83 (0.63–1.09)	OR: 0.75 (0.57–1.00)	OR: 0.42 (0.30–0.59)					
								*p* = 0.17	*p* = 0.05	*p* < 0.0001					
						Marital status	Married	Not married							
							384/957	225/658							
							(40.1)	(34.2)							
							OR: Ref	OR: 0.78 (0.63–0.95)							
								*p* = 0.02							
						Education	College+	Some college	HS	< HS					
							279/687	181/474	112/333	37/121					
							(40.6)	(38.2)	(33.6)	(30.6)					
							OR: Ref	OR: 0.90 (0.71–1.15)	OR: 0.74 (0.56–0.97)	OR: 0.64 (0.43–0.98)					
								*p* = 0.41	*p* = 0.03	*p* = 0.04					
						Insurance	Work	Medicare	Medicaid	Self/other		None			
							357/893	126/351	59/198	32/76		35/97			
							(40.0)	(35.9)	(29.8)	(42.1)		(36.1)			
							OR: Ref	OR: 0.84 (0.65–1.09)	OR: 0.64 (0.46–0.89)	OR: 1.09 (0.68–1.76)		OR: 0.85 (0.55–1.31)			
								*p* = 0.18	*p* = 0.008	*p* = 0.72		*p* = 0.46			
						Individual SES	High SES/low comorbid	Low SES/high comorbid							
							367/897	242/718							
							(40.9)	(33.7)							
							OR: Ref	OR: 0.73 (0.60–0.90)							
								*p* = 0.003							
Zipkin et al. (2020) [36]^d^	USA	Centers for Medicare and Medicaid Services (CMS) Master Beneficiary Summary, Medicare Provider Analysis and Review, Carrier, and Outpatient Services files, Dartmouth Atlas Health	Breast cancer *n* = 156,229	Oncotype DX *n* = 18,244 (11.7)	No testing	Age at diagnosis (years)	65–69	70–75	76–79	80+				< 0.001	7.5
							7560/42,256	6165/40,208	3190/33,198	1329/40,567					
							(17.9)	(15.3)	(9.6)	(3.3)					
							OR: Ref	OR: 0.83 (0.80–0.86)	OR: 0.49 (0.47–0.51)	OR: 0.16 (0.15–0.17)					
								*p* < 0.0001	*p* < 0.0001	*p* < 0.0001					
						Race	White	Black	Other					< 0.001	
							16,735/140,397	1005/11,055	504/4777						
							(11.9)	(9.1)	(10.6)						
							OR: Ref	OR: 0.74 (0.69–0.79)	OR: 0.87 (0.79–0.96)						
								*p* < 0.0001	*p* = 0.004						
						Treated at teaching hospital	Yes	No						0.084	
							4551/38,160	13,693/118,069							
							(11.9)	(11.6)							
							OR: Ref	OR: 0.97 (0.93–1.004)							
								*p* = 0.08							
						Rural	Yes	No						< 0.001	
							4038/32,797	14,206/123,432							
							(12.3)	(11.5)							
							OR: Ref	OR: 0.93 (0.89–0.96)							
								*p* = 0.0001							
NGS Testing: all tumour types															
Bruno et al. (2022)^d^, ^f^ [37]	USA	Flatiron Health Database	All tumours *n* = 28,256	NGS assay (any, not specified) *n* = 13,239 (46.9)	No testing OR biomarker testing (other)	Ethnicity (all tumour types)	White	Black							7.5
							11,836/24,615	1,403/3,641							
							(48.1)	(38.5)							
							OR: Ref	OR: 0.68 (0.63–0.73)							
								*p* < 0.0001							
						Ethnicity NSCLC *n* = 11,081	White	Black						< 0.0001	
							4904/9793	513/1288							
							(50.1)	(39.8)							
							OR: Ref	OR: 0.66 (0.59–0.74)							
								< 0.0001							
						Ethnicity non‐squamous NSCLC *n* = 7627	White	Black						< 0.0001	
							3668/6705	404/922							
							(54.7)	(43.8)							
							OR: Ref	OR: 0.65 (0.56–0.74)							
								< 0.0001							
						Ethnicity colorectal cancer *n* = 5641	White	Black						< 0.0001	
							2478/4803	350/838							
							(51.6)	(41.8)							
							OR: Ref	OR: 0.67 (0.58–0.78)							
								< 0.0001							
						Ethnicity breast cancer *n* = 3907	White	Black						0.68	
							786/3314	136/593							
							(23.7)	(22.9)							
							OR: Ref	OR: 0.96 (0.78–1.18)							
								*p* = 0.68							
Bruno et al. (2024) [38]^d^	USA	Market Scan Research Multi‐State Medicaid Database	Lung cancer *n* = 3845	NGS assay not specified *n* = 166 (4.3)	No testing	Race or ethnicity^h^	Black	White	Other	Hispanic		Unknown		0.28 (Black vs. White vs, Other)	7
							34/970	102/2271	3/122	3/57		24/425			
							(3.5)	(4.5)	(2.5)	(5.3)		(5.6)			
							OR: 0.77 (0.52‐1.15)	OR: Ref	OR: 0.54 (0.17–1.72)	OR: 1.18 (0.36–3.84)		OR: 1.27 (0.81–2.01)			
							*p* = 0.20		*p* = 0.29	*p* = 0.78		*p* = 0.30			
Chehade et al. (2024) [39]	USA	Flatiron Health Database	Metastatic prostate cancer *n* = 11,927	NGS assay not specified	No testing	Socioeconomic status	1 (Lowest)	2	3	4		5 (Highest)			7
							HR: 0.74 (0.66–0.83)	(0.80–0.99)	HR: 0.90 (0.82–1.00)	HR: 0.93 (0.84–1.03)		HR: Ref			
							*p* < 0.001	*p* = 0.03	*p* = 0.05	*p* = 0.14					
						Race and ethnicity	White	Asian	Black	Hispanic or Latino		Other			
							HR: Ref	HR: 0.84 (0.63–1.11)	HR: 0.75 (0.67–0.84)	HR: 0.70 (0.60–0.82)		HR: 0.97 (0.88–1.07)			
								*p* = 0.22	*p* < 0.001	*p* < 0.001		*p* = 0.54			
						Insurance	Commercial	Medicare or other government program	Medicaid	Other					
							HR: Ref	HR: 0.89 (0.82–0.98)	HR: 0.53 (0.38–0.74)	HR: 1.10 (0.97–1.25)					
								*p* = 0.01	*p* < 0.001	*p* = 0.13					
			Advanced urothelial carcinoma *n* = 6490	NGS assay not specified	No testing	Socioeconomic status	1 (Lowest)	2	3	4		5 (Highest)			
							HR: 0.77 (0.66–0.89)	HR: 0.87 (076–1.00)	HR: 0.97 (0.86–1.11)	HR: 0.95 (0.84–1.07)		HR: Ref			
							*p* < 0.001	*p* = 0.049	*p* = 0.69	*p* = 0.40					
						Race and ethnicity	White	Asian	Black	Hispanic or Latino		Other			
							HR: Ref	HR: 1.06 (0.75–1.50)	HR: 0.76 (0.61–0.96)	HR: 0.88 (0.70–1.10)		HR: 1.08 (0.96–1.22)			
								*p* = 0.73	*p* = 0.02	*p* = 0.18		*p* = 0.18			
						Insurance	Commercial	Medicare or other government program	Medicaid	Other					
							HR: Ref	HR: 0.88 (0.78–0.99)	HR: 0.72 (0.53–0.97)	HR: 1.06 (0.91–1.23)					
								*p* = 0.03	*p* = 0.03	*p* = 0.47					
Halder et al. (2022)^d^ [40]	USA, Arizona	Arizona Cancer Centre Database	Pancreatic cancer *n* = 198	NGS assay not specified *n* = 97 (49.0)	No testing	Ethnicity	Hispanic	Other—Non‐Hispanic						0.298	8
							17/41 (41.5)	80/157 (51.0)							
							OR: 0.68 (0.34–1.37)	OR: Ref							
							*p* = 0.28								
Huang et al. (2019)^d^ [41]	USA, Florida	University of Miami Hospitals Tumour Registry	Gynaecological cancers *n* = 367	Caris Molecular Intelligence or Foundation Medicine *n* = 99 (27.0)	No testing	Age^b^ (years)	< 65	≥ 65						0.751	10
							67/253	32/144							
							(26.5)	(28.1)							
						Race/ethnicity	Black	Hispanic White	Non‐Hispanic White	Other/unknown				0.425	
							11/40	40/166	45/144	3/17					
							(27.5)	(24.1)	(31.3)	(17.6)					
							OR: 0.83 (0.38–1.81)	OR: 0.70 (0.42–1.15)	OR: Ref	OR: 0.47 (0.13–1.72)					
							*p* = 0.65	*p* = 0.16		*p* = 0.26					
						Insurance	Medicaid	Medicare	Private	Self‐pay	Uninsured			< 0.001	
							2/60	26/101	71/182	0/4	0/20				
							(3.3)	(25.7)	(39.0)	(0)	(0)				
							OR: 0.15 (0.04–0.62)	OR: 0.54 (0.28–1.03)	OR: Ref	OR: 0.2 (0.0–8.25)	OR: 0.25 (0.01–6.13)				
							*p* = 0.0009	*p* = 0.061		*p* = 0.394	*p* = 0.397				
						Type of hospital	Comprehensive Cancer Centre	Safety Net Hospital						< 0.001	
							97/283	2/84							
							(34.3)	(2.4)							
							OR: 5.78 (1.35–24.76)	OR: Ref							
							*p* = 0.018								
Hasson et al. (2022)^d^ [42]	Israel	Tel‐Aviv Sourasky Medical Centre Database	Ovarian Cancer *n* = 1026	Foundation One CDx GCP Tissue *n* = 108 (10.5)	No testing	Age (years)	Received test median: 62.7	Did not receive test (control) median: 61.3						0.373	6.5
						Ethnicity	Ashkenazi Jewish:	Not Ashkenazi Jewish (control)						0.0007	
							75/108	422/832							
							(69.4)	(50.7)							
							OR: Ref	OR: 0.45 (0.29–0.70)							
								*p* = 0.0003							
Kehl et al. (2019) [43]	USA	Surveillance, Epidemiology, and End Results Programme (SEER)‐Medicate	Stage IV Lung Adenocarcinoma *n* = 5556	NGS assay not specified *n* = 1439 (25.9)	No testing	Age at diagnosis (years)	66–70	71–75	76–80	81–85	86–99			0.04	7.5
							394/1516	396/1485	296/1187	248/877	104/491				
							(26.0)	(26.7)	(24.9)	(28.3)	(21.2)				
							OR: Ref	OR: 0.98 (0.82–1.17)	OR: 0.91 (0.75–1.10)	OR: 1.15 (0.94–1.42)	OR: 0.75 (0.58–0.99)				
						Sex	Male	Female						0.002	
							579/2402	858/3154							
							(24.1)	(27.2)							
							OR: Ref	OR: 1.25 (1.08–1.44)							
						Race	White	Black	Asian/other					< 0.001	
							1174/4482	66/467	199/607						
							(26.2)	(14.1)	(32.8)						
							OR: Ref	OR: 0.53 (0.40–0.72)	OR: 1.54 (1.23–1.93)						
						Ethnicity	Non‐Hispanic	Hispanic						0.52	
							1357/5198	81/358							
							(26.1)	(22.6)							
							OR: Ref	OR: 0.91 (0.68–1.22)							
						Medicaid dual‐eligible	No	Yes						0.01	
							1068/3761	370/1795							
							(28.4)	(20.6)							
							OR: Ref	OR: 0.79 (0.67–0.95)							
						Census tract‐level poverty rate (quintile)	0% (1)	> 0% to 4.3% (2)	4.3% to 8.5% (3)	8.5% to 15.8% (4)	> 15.8% (5)			0.18	
							347/1131	298/1091	294/1112	277/1111	221/1111				
							(30.7)	(27.3)	(26.4)	(24.9)	(19.9)				
							OR: Ref	OR: 0.91 (0.75–1.12)	OR: 0.96 (0.79–1.18)	OR: 0.94 (0.77–1.16)	OR: 0.77 (0.61–0.96)				
						Urban‐rural code	Large metro	Metro	Urban	Less urban	Rural			< 0.001	
							858/3044	372/1520	78/324	107/550	21/118				
							(28.2)	(24.5)	(24.1)	(19.5)	(17.8)				
							OR: Ref	OR: 0.81 (0.69–0.94)	OR: 0.76 (0.57–1.02)	OR: 0.59 (0.46–0.76)	OR: 0.59 (0.35–0.98)				
						Marital status	Married/partnered	Unmarried	Unknown					0.10	
							735/2587	792/2,770	46/199						
							(28.4)	(28.6)	(23.3)						
							OR: Ref	OR: 0.85 (0.74–0.99)	OR: 0.90 (0.64–1.28)						
						Poor disability status	No	Yes						< 0.001	
							1336/4876	100/680							
							(27.4)	(14.7)							
							OR: Ref	OR: 0.61 (0.48–0.79)							
						Care at National Cancer Institute Centre	No	Yes						< 0.001	
							1174/4912	261/644							
							(23.9)	(40.5)							
							OR: Ref	OR: 1.96 (1.62–2.36)							
Khan et al. (2024) [44]^d^	USA	Medicare Standard Analytic Files	Gastrointestinal/Lung/or Breast Cancer *n* = 1,466,105	NGS assay not specified *n* = 26,608 (1.8)	No testing	Sex	Male	Female						< 0.001	9
							10,853/539,201	15,755/926,904							
							(2.01)	(1.70)							
							OR: Ref	OR: 0.84 (0.82–0.86)							
								*p* < 0.0001							
						Race	Non‐Hispanic White	Non‐Hispanic Black	Hispanics	Non‐Hispanic Other				< 0.001	
							23,687/1,287,805	1381/105,476	133/12,984	1407/59,840					
							(1.84)	(1.31)	(1.02)	(2.35)					
							OR: Ref	OR: 0.71 (0.67–0.75)	OR: 0.55 (0.47–0.66)	OR: 1.29 (1.22–1.36)					
								*p* < 0.0001	*p* < 0.0001	*p* < 0.0001					
						Metropolitan	Metropolitan	Non‐Metropolitan						< 0.001	
							22,133/1,142,697	4475/323,408							
							(1.94)	(1.38)							
							OR: Ref	OR: 0.71 (0.69–0.73)							
								*p* < 0.0001							
						Social Vulnerability Index	Low	Moderate	High					< 0.001	
							9594/489,345	8836/498,932	8178/477,828						
							(1.96)	(1.77)	(1.71)						
							OR: Ref	OR: 0.90 (0.88–0.93)	OR: 0.87 (0.85–0.90)						
								*p* < 0.0001	*p* < 0.0001						
Markt et al.^g^ (2022) [45]	USA	Flatiron Health Database	Metastatic Colorectal Cancer *n* = 25,469	NGS assay not specified *n* = 3,130 (12.3)	No testing	Age	60–69	70–79	80+						9
							OR Ref	OR: 0.74 (0.65–0.85)	OR: 0.86 (0.72–1.03)						
								*p* < 0.01	*p* = 0.10						
						Sex	Male	Female							
							OR: Ref	OR: 0.97 (0.87–1.09)							
								*p* = 0.65							
						Race	White	Black	Asian	Hispanic	Other	Missing			
							OR: Ref	OR: 0.73 (0.59–0.9)	OR: 0.85 (0.57–1.24)	OR: 1.24 (0.94–1.62)	OR: 1.05 (0.86–1.27)	OR: 1.12 (0.92–1.35)			
								*p* < 0.01	*p* = 0.41	*p* = 0.13	*p* = 0.64	*p* = 0.27			
						Insurance	Commercial Health Plan	Medicare	OGP	Unknown					
							OR: Ref	OR: 0.83 (0.69–1.0)	OR: 0.84 (0.63–1.1)	OR: 0.79 (0.69–0.9)					
								*p* = 0.05	*p* = 0.21	*p* < 0.01					
Meernik et al. (2024)^d^ [46]	USA, North Carolina	Duke Cancer Institute multilevel data warehouse (DCI CREST)	Stage IV Breast, Colorectal, NSCLC or Prostate Cancer *n* = 3,461	Any genomic testing *n* = 1,541 (44.5)	No testing	Race and ethnicity^h^	Hispanic	NH Asian	NH Black	NH White	NH Other races	Unknown			9.5
							21/49	47/71	358/882	1065/2322	21/50	29/87			
							(42.9)	(66.2)	(40.6)	(45.9)	(42.0)	(33.3)			
							OR: 0.89 (0.50–1.57)	OR: 2.31 (1.40–3.81)	OR: 0.81 (0.69–0.94)	OR: Ref	OR: 0.85 (0.48–1.51)	OR: 0.59 (0.38–0.93)			
							*p* = 0.68	*p* = 0.001	*p* = 0.007		*p* = 0.59	*p* = 0.02			
						Sex	Male	Female							
							798/1945	743/1516							
							(41.0)	(49.0)							
							OR: Ref	OR: 1.38 (1.21–1.58)							
								*p* < 0.0001							
						Insurance	Private	Medicaid	Medicare	Other insurance	Uninsured				
							531/1026	86/204	708/1627	183/517	33/87				
							(51.8)	(42.2)	(43.5)	(35.4)	(37.9)				
							OR: Ref	OR: 0.68 (0.50–0.92)	OR: 0.72 (0.61–0.84)	OR: 0.51 (0.41–0.64)	OR: 0.57 (0.36–0.89)				
								*p* = 0.01	*p* < 0.0001	*p* < 0.0001	*p* = 0.01				
						Rural‐urban	Isolated small rural	Small rural	Large rural/city/town	Urban	Unknown				
							1106/2463	178/395	122/244	19/61	116/298				
							(44.9)	(45.1)	(50.0)	(31.1)	(38.9)				
							OR: 0.82 (0.63–1.06)	OR: 0.82 (0.60–1.13)	OR: Ref	OR: 0.45 (0.25–0.82)	OR: 0.64 (0.45–0.90)				
							*p* = 0.13	*p* = 0.22		*p* = 0.009	*p* = 0.01				
						Proportion of census tract with ≤ high school education, quintiles	1 (lowest proportion low education)	2	3	4	5	Unknown			
							234/584	174/417	238/540	325/689	437/903	133/328			
							(40.1)	(41.7)	(44.1)	(47.2)	(48.4)	(40.5)			
							OR: 0.71 (0.58–0.88)	OR: 0.76 (0.60–0.97)	OR: 0.84 (0.68–1.04)	OR: 0.95 (0.78–1.16)	OR: Ref	OR: 0.73 (0.56–0.94)			
							*p* = 0.002	*p* = 0.02	*p* = 0.11	*p* = 0.63		*p* = 0.01			
						Yost SES index, quintiles	1 (lowest SES)	2	3	4	5 (highest SES)	Unknown			
							374/750	294/684	359/738	241/609	156/381	117/299			
							(49.9)	(43.0)	(48.6)	(39.6)	(40.9)	(39.1)			
							OR: 1.43 (1.12–1.84)	OR: 1.09 (0.84–1.40)	OR: 1.37 (1.06–1.75)	OR: 0.94 (0.73–1.23)	OR: Ref	OR: 0.93 (0.68–1.26)			
							*p* = 0.005	*p* = 0.52	*p* = 0.01	*p* = 0.67		*p* = 0.63			
Presley et al. (2018)^d^ [47]	USA	Flatiron Health Database	Advanced NSCLC *n* = 5,688	NGS assay (multiple) *n* = 875 (15.4)	No testing	Age (years)	≤ 45	46–55	56–65	66–75	76–85			< 0.001	8
							41/130	106/694	277/1644	307/1949	144/1271				
							(31.5)	(15.3)	(16.8)	(15.8)	(11.3)				
							OR: Ref	OR: 0.39 (0.26–0.60)	OR: 0.44 (0.30–0.65)	OR: 0.41 (0.28–0.60)	OR: 0.28 (0.18–0.42)				
								*p* < 0.0001	*p* < 0.0001	*p* < 0.0001	*p* < 0.0001				
						Race/ethnicity	Non‐Hispanic White	Non‐Hispanic Black	Hispanic or Latino	Asian	Other	Unknown		< 0.001	
							594/3617	49/429	28/194	39/211	98/475	67/762			
							(16.4)	(11.4)	(14.4)	(18.5)	(20.6)	(8.8)			
							OR: Ref	OR: 0.66 (0.48–0.90)	OR: 0.86 (0.67–1.29)	OR: 1.15 (0.81–1.65)	OR: 1.32 (1.04–1.68)	OR: 0.49 (0.38–0.64)			
								*p* = 0.008	*p* = 0.47	*p* = 0.43	*p* = 0.02	*p* < 0.0001			
						Sex	Male	Female						0.93	
							407/2654	468/3034							
							(15.3)	(15.4)							
							OR: Ref	OR: 1.01 (0.87–1.16)							
								*p* = 0.93							
						Insurance	Commercial	Medicare	Medicaid	Other payer	Unknown			0.10	
							364/2142	146/1058	7/60	116/762	242/1666				
							(17.0)	(13.8)	(11.7)	(15.2)	(14.5)				
							OR: Ref	OR: 0.78 (0.63–0.96)	OR: 0.65 (0.29–1.43)	OR: 0.88 (0.70–1.10)	OR: 0.83 (0.70–0.99)				
								*p* = 0.02	*p* = 0.28	*p* = 0.26	*p* = 0.04				
						Income, by Quintile	1	2	3	4	5	Unknown		< 0.001	
							71/555	95/732	163/1090	177/1214	357/1969	12/128			
							(12.8)	(13.0)	(15.0)	(14.6)	(18.1)	(9.4)			
							OR: 0.66 (0.50–0.87)	OR: 0.67 (0.53–0.86)	OR: 0.79 (0.65–0.97)	OR: 0.77 (0.63–0.94)	OR: Ref	OR: 0.47 (0.26–0.86)			
							*p* = 0.003	*p* = 0.002	*p* = 0.03	*p* = 0.009		*p* = 0.01			
						Smoking^h^	No history of smoking	History of smoking	Unknown					< 0.001	
							> 218/> 1044	652/4543	< 5/< 101						
							(20.9)	(13.4)	(5.0)						
							OR: Ref	OR: 0.63 (0.54–0.75)	OR: 0.20 (0.08–0.49)						
								*p* < 0.0001	*p* = 0.0005						
Tuminello et al. (2024) [48]	USA	National Cancer Institutes' Surveillance, Epidemiology and End Results (SEER) population‐based Cancer Registry Program	NSCLC all stages *n* = 28,511. Subgroup analysis of Stage IV only *n* = 11,538	Molecular diagnostic test incl. NGS assays (multiple) *n* = 11,209 (39.3)	No testing	Race	Black	Other	White					< 0.001	9.5
							644/2310	661/1687	9904/24,514						
							(27.9)	(39.2)	(38.8)						
							OR: 0.64 (0.58–0.71)	OR: 0.92 (0.83–1.02)	OR: Ref						
						Race (Stage IV only)	OR: 0.58 (0.50–0.67)	OR: 0.88 (0.75–1.02)	OR: Ref						
						Census Tract Poverty Indicator, % in poverty	< 10	> 10						< 0.001	
							5506/13,055	5694/15,436							
							(42.2)	(36.9)							
							OR: Ref	OR: 0.85 (0.80–0.89)							
						Census Tract Poverty Indicator, % in poverty (Stage IV only)	OR: Ref	OR: 0.82 (0.76–0.89)							
						Residence	Metro, > 20 thousand	Urban, 2.5–20 thousand	Rural, < 2.5 thousand					0.019	
							9044/23,243	1809/4,398	268/667						
							(38.9)	(41.1)	(40.2)						
							OR: Ref	OR: 1.15 (1.07–1.23)	OR: 1.14 (0.97–1.34)						
						Residence (Stage IV only)	OR: Ref	OR: 1.19 (1.07–1.32)	OR: 1.31 (1.03–1.67)						
						Sex	Male	Female						< 0.001	
							5448/14,364	5761/14,147							
							(37.9)	(40.7)							
							OR: Ref	OR: 1.14 (1.08–1.20)							
						Sex (Stage IV only)	OR: Ref	OR: 1.10 (1.01–1.19)							
						Age at diagnosis (years)	65–69	70–74	75–79	80–84	85 and older			0.49	
							2273/5889	3131/7891	2685/6719	1859/4759	1261/3253				
							(38.6)	(39.7)	(40.0)	(39.1)	(38.8)				
							OR: Ref	OR: 1.06 (0.98–1.13)	OR: 1.07 (0.99–1.15)	OR: 1.04 (0.96–1.12)	OR: 1.04 (0.95–1.14)				
						Age at diagnosis (years) (Stage IV only)	OR: Ref	OR: 1.08 (0.97–1.20)	OR: 1.07 (0.95–1.20)	OR: 0.98 (0.86–1.11)	OR: 0.98 (0.85–1.13)				
						Marital status	Single	Married	Unknown					< 0.001	
							4799/13,067	5994/14,224	416/1220						
							(36.7)	(42.1)	(34.1)						
							OR: Ref	OR: 1.24 (1.18–1.31)	OR: 0.92 (0.81–1.05)						
						Marital status (Stage IV only)	OR: Ref	OR: 1.36 (1.26–1.48)	OR: 0.91 (0.74–1.13)						
Zhao et al. (2024) [49]^j^	USA	Mayo Clinic Health System	All tumours *n* = 9886. Lung Cancer only subgroup analysis *n* = 2231	Large sized panel genetic test all tumours (50+ genes) *n* = 2909 (29.4). Medium sized panel genetic test all tumours (2–49 genes) *n* = 2384 (24.1). Large sized panel genetic test lung cancer (50+ genes) *n* = 390 (17.5). Medium sized panel genetic test lung cancer (2–49 genes) *n* = 1294 (58.0)	Single gene testing	Race	White	Asian/Pacific Islander	African American	Hispanic/Latino	Other (incl. American Indian)	Unknown			9.5
							Large	Large	Large	Large	Large	Large			
							2478/8193	86/200	91/168	98/233	64/190	92/902			
							(30.2)	(43.0)	(54.2)	(42.1)	(33.7)	(10.2)			
							Medium	Medium	Medium	Medium	Medium	Medium			
							2002/8193	38/200	16/168	30/233	40/190	258/902			
							(24.4)	(19.0)	(9.5)	(12.9)	(21.1)	(28.6)			
							Single	Single	Single	Single	Single	Single			
							3713/8193	76/200	61/168	105/233	86/190	552/902			
							(45.3)	(38.0)	(36.3)	(45.1)	(45.3)	(61.2)			
						Sex	Male	Female	Missing						
							Large	Large	Large						
							1405/4596	1465/4549	39/741						
							(30.6)	(32.2)	(5.3)						
							Medium	Medium	Medium						
							1194/4596	974/4549	216/741						
							(26.0)	(21.4)	(29.1)						
							Single	Single	Single						
							1997/4596	2110/4549	486/741						
							(43.5)	(46.4)	(65.6)						
					
						Area Deprivation Index	Low (values 1–3)	Medium (4–6)	High (7–10)	Unknown					
							Medium + Large vs. Single gene	Medium + Large vs. Single gene	Medium + Large vs. Single gene	Medium + Large vs. Single gene					
							1271/2056	999/1741	849/1621	2174/4468					
							(61.8)	(57.4)	(52.4)	(48.7)					
							OR: Ref	OR: 0.86 (0.74–0.99)	OR: 0.71 (0.61–0.83)						
								*p* = 0.035	*p* < 0.001						
							Large vs. Single gene	Large vs. Single gene	Large vs. Single gene	Large vs. Single gene					
							890/1,675	561/1,303	404/1,176	1,054/3,348					
							(53.1)	(43.1)	(34.4)	(31.5)					
							OR: Ref	OR: 0.75 (0.64–0.89)	OR: 0.58 (0.49–0.69)						
								*p* = 0.001	*p* < 0.001						
						Area Deprivation Index—Lung Cancer Only	Low (values 1–3)	Medium (4–6)	High (7–10)						
							Medium + Large vs. Single gene	Medium + Large vs. Single gene	Medium + Large vs. Single gene						
							OR: Ref	OR: 0.90 (0.64–1.26)	OR: 1.00 (0.71–1.41)						
								*p* = 0.536	*p* = 0.989						
							Large vs. Single gene	Large vs. Single gene	Large vs. Single gene						
							OR: Ref	OR: 0.75 (0.49–1.15)	OR: 0.76 (0.48–1.20)						
								*p* = 0.187	*p* = 0.240						
						Rural/Urban^k^—Lung Cancer Only	Urban	Rural	Unknown						
							Medium + Large vs. Single gene	Medium + Large vs. Single gene	Medium + Large vs. Single gene						
							2540/4544	1204/2313	549/3029						
							(55.9)	(52.1)	(51.1)						
							OR: Ref	OR: 0.85 (0.76–0.96)							
								*p* = 0.006							
							Large vs. Single gene	Large vs. Single gene	Large vs. Single gene						
							1605/3609	476/1585	828/2308						
							(44.5)	(30.0)	(35.9)						
							OR: Ref	OR: 0.54 (0.47–0.63)							
	*p* < 0.001														
					
						Rural/Urban—Lung Cancer Only	Urban	Rural							
							Medium + Large vs. Single gene	Medium + Large vs. Single gene							
							OR: Ref	OR: 0.93 (0.73–1.21)							
								*p* = 0.638							
							Single gene	Large vs. Single gene							
							OR: Ref	OR: 0.44 (0.30–0.65)							
	*p* < 0.001														

Abbreviations: ER, endocrine receptor; HER2, human epidermal growth factor Receptor 2; HR, hormone receptor; NGS, Next Generation Sequencing; NSCLC, non‐small cell lung cancer; SES, socioeconomic status.

^a^ Refers to total number of patients in the cohorts of interest.

^b^ Comparable adjusted OR not available in study and cannot be calculated from raw data.

^c^ Area‐based composite socioeconomic status was based on the Yost Index using US 2010 Census Tract data.

^d^ Author generated OR.

^e^ All subcategory numbers expressed as percentages only in raw data.

^f^ Combined cohort numbers calculated by the authors. Black and white ethnicity utilisation only reported by Bruno et al [37].

^g^ Raw data not available from study, OR only.

^h^ Presumed to be exact numbers for OR calculations.

^i^ Race/ethnicity data omitted as study reported in Dunn et al [28].

^j^ Adjusted ORs not provided for Race and Sex.

^k^ Adjusted ORs not provided for Unknown Rural/Urban residence.

### 3.2 Study Characteristics

The included 24 studies covered two main testing modalities: Oncotype DX (*n* = 11 studies) [14, 27, 28, 29, 30, 31, 32, 33, 34, 35, 36] and NGS sequencing technology (*n* = 13) [37, 38, 39, 40, 41, 42, 43, 44, 45, 46, 47, 48, 49]. Foundation One panels represented the most common NGS sequencing modalities named by included studies (*n* = 2) [41, 42]. Included NGS studies detailed six main primary tumour sites (non‐small cell lung cancer (NSCLC), gastrointestinal, gynaecological malignancies, pancreatic, prostate and breast cancer). Most studies were from the USA (*n* = 23) [14, 27, 28, 29, 30, 31, 32, 33, 34, 35, 36, 37, 38, 39, 40, 41, 43, 44, 45, 46, 47, 48, 49] with the remaining study from Israel [42]. Three studies on NGS reported data from the Flatiron Database in three tumour types—urological, colorectal and NSCLC, respectively [39, 45, 47]. One study from the Flatiron Database reported breast cancer in addition to colorectal and NSCLC data as one combined cohort [37]. Two studies on NGS utilisation reported data on NSCLC from the National Cancer Institutes' Surveillance, Epidemiology and End Results (SEER) population based Cancer Registry Programme [43, 48]. Three Breast Cancer Oncotype DX studies reported data from the National Cancer Database [14, 30, 34]. These reported data across differing stages and types of breast cancer with socio‐demographic factors defined by different subcategories (e.g., inclusion of Location: metro/urban/rural [14]; or education status [34]). Two Oncotype DX studies reported data from The Carolina Breast Cancer Study Cohort [28, 35].

Socio‐demographic measures of test utilisation were reported across 12 measures: race and/or ethnicity (*n* = 23) [14, 27, 28, 29, 30, 31, 32, 33, 34, 36, 37, 38, 39, 40, 41, 42, 43, 44, 45, 46, 47, 48, 49] age (*n* = 16) [14, 27, 29, 30, 31, 32, 33, 34, 35, 36, 41, 42, 43, 45, 47, 48]; insurance status (*n* = 15) [14, 27, 29, 30, 31, 32, 33, 34, 35, 39, 41, 43, 45, 46, 47]; socio‐economic status (*n* = 13) [14, 27, 31, 33, 34, 35, 39, 43, 44, 46, 47, 48, 49]; sex (*n* = 8) [29, 43, 44, 45, 46, 47, 48, 49]; location (urban/rural, etc.) (*n* = 7) [14, 36, 43, 44, 46, 48, 49]; marital status (*n* = 5) [31, 33, 35, 43, 48]; education (*n* = 3) [34, 35, 46]; and hospital status (e.g., teaching hospital) (*n* = 3) [36, 41, 43]. Three socio‐demographic measures were only reported in one study each, these included—smoking status [47]; distance from large centre [29]; and disability status [43]. Four studies [28, 37, 38, 40] reported socio‐demographic information by one measure, race and/or ethnicity, only; the others all reported multiple measures. For one study [33] utilisation was available as a percentage only. One study reported hazard ratios (HRs), confidence intervals (CIs) and *p*‐values only [39]. The majority of studies (*n* = 23) featured no testing as a comparator, with one study comparing utilisation by size of panel (large and medium) against single gene testing [49]. No paediatric specific studies were retrieved.

### 3.3 Quality Appraisal

The 24 included studies quality scores using a modified ISPOR checklist ranged from 6.5 to 10 out of a possible 10 (mean = 8.3, median 8) (Table S5). The study populations, socio‐demographic factors, and numerical reporting were often described well in the included studies. Methods for adjusting for confounders and statistical analysis for associations between exposure (socio‐demographic metric) and outcomes (test utilisation) scored lower.

### 3.4 Socio‐Demographic Factors and Utilisation of Oncotype DX

The data reported in studies on Oncotype DX testing in both breast and prostate cancer was collected from 2004 to 2018. Increasing age was significantly associated with decreased likelihood of utilisation of Oncotype DX across seven of ten studies which reported age [27, 29, 30, 32, 34, 35, 36]. Race and ethnicity were reported across a diverse range of classifications. In seven of ten studies reporting on race and/or ethnicity—white or non‐Hispanic white was associated with an increased likelihood of receipt of testing [28, 30, 31, 32, 33, 34, 36]. Likewise, privately insured patients or fee‐based care (as opposed to Medicaid, Medicare, uninsured or non‐fee‐based care) were more likely to be in receipt of testing; this was reported in seven of eight studies which reported on insurance status [14, 30, 31, 32, 33, 34, 35].

Socio‐economic status (median income and education level) was reported in six studies; five demonstrated that increasing socio‐economic status was related to higher receipt of testing [14, 27, 31, 33, 35]. However, one study reported increased utilisation with lower socio‐economic status (based on median income) [34] as part of an adjusted OR model. Three studies reported on marital status, with two of them finding being single/unmarried or divorced/separated associated with a higher likelihood of Oncotype DX testing when compared to married patients [30, 31]. In one such study, widowed patients had a significantly reduced likelihood of testing (OR 0.79, 95% CI 0.69–0.89) [33]. As the breast cancer Oncotype DX testing modality is female predominant, only one study reported on utilisation by sex, in US veterans. This found low Oncotype DX utilisation in male compared to female breast cancer; however, the number of cases in males was small and the association was not statistically significant (*n* = 72/267 and 10/61 respectively, OR 0.53, 95% CI 0.26–1.10, *p* = 0.09) [29].

### 3.5 Socio‐Demographic Factors and Utilisation of NGS Testing

Thirteen included studies presented data on NGS testing across six main tumour types; pancreatic (*n* = 1) [40], prostate (*n* = 2) [39, 46], gastrointestinal (*n* = 4) [37, 44, 45, 46], gynaecological (*n* = 2) [41, 42], NSCLC (*n* = 8) [37, 38, 43, 44, 46, 47, 48, 49] and breast (*n* = 3) [37, 44, 46]. Data presented was collected from 2002 to 2023. All thirteen studies included reported race and/or ethnicity and nine studies showed an association with non‐white races and/or ethnicities and a decreased likelihood of receipt of NGS [37, 38, 39, 40, 41, 43, 44, 46, 47]. One study [42] reported ORs based on Ashkenazi Jewish origin (no vs. yes) and demonstrated a decreased likelihood of testing in patients not of Ashkenazi Jewish origin in Israel (OR 0.45, 95% CI 0.29–0.70, *p* = 0.0003). Three studies, in US populations, identified increased ORs for utilisation of testing in Asian populations when compared to white populations (OR: 1.54 [1.23–1.93] [43]; OR: 2.31 [1.40–3.81] [46]; OR: 1.15 [0.81–1.65] [47]). These studies all reported on populations exclusively consisting of or containing patients with NSCLC. Age was reported in five studies [41, 42, 43, 45, 47] and in two studies older age was associated with decreased utilisation [45, 47]. Two studies did not report ORs for age [41, 42]. One study [43] demonstrated inconsistency across age ranges reported, from 66 to 99 years old, with increased utilisation between ages 81–85 years old when compared to 66–70 years old (OR: 1.15 [0.94–1.42]).

Insurance status was reported in six studies [39, 41, 43, 45, 46, 47]; private insurance (as opposed to Medicaid, Medicare or uninsured) was associated with increased receipt of testing in all six studies. Socio‐economic status was reported in seven studies and lower socio‐economic status was associated with decreased utilisation in six of these [39, 43, 44, 47, 48, 49]. Sex was reported in seven studies [43, 44, 45, 46, 47, 48, 49]. Female sex was associated with increased utilisation in four studies on patients with NSCLC or containing patients with NSCLC [43, 46, 47, 48]. In one NSCLC study [47] a history of smoking was associated with reduced likelihood of testing when compared to never smokers (OR 0.63 [0.54–0.75], *p* < 0.001). Disability status was reported by one study. This found significantly decreased test utilisation in patients with advanced lung cancer and a poor disability status (OR: 0.61 [0.48–0.79], *p* < 0.001) [43].

## 4 Discussion

This systematic review represents the first to assess utilisation of novel tumour and ctDNA somatic mutation testing in solid tumour by socio‐demographic factors since 2018. The review serves as a timely update as significant advances in access to testing have been made in recent years. Across multiple tumour types, testing modalities and socio‐demographic characteristics, statistically significant disparities were identified. These were most comprehensively characterised by decreased test utilisation in: non‐white races and ethnicities; older age; those with non‐private insurance statuses; and less socio‐economically advantaged groups. These findings support the previous literature which has highlighted inequities across the cancer treatment pathway [1, 2, 3, 4, 6] as well as older reviews exploring disparities in access to novel biomarker guided therapy [5]; access to treatment; and survival [51].

Studies on the Oncotype DX testing panels in breast and prostate cancer represented slightly less than half of those identified. As a more established testing modality at time of publication [11], it was of interest to assess if disparities in access were present following over a decade of usage and familiarisation. Literature prior to inclusion criteria dates for this review had consistently identified disparities in Oncotype DX utilisation by race [52, 53, 54]; increasing age [55]; insurance status and reimbursement of test cost [56]. It might have been expected that, as both clinicians and patients became more familiar with the test, and it became more widely used, inequalities might disappear over time. In fact, the contemporary literature identified by the present review found broadly the same patterns, suggesting a lack of significant shift in the identified inequalities in the intervening time period. Indeed, inequities of access by race; socioeconomic group and location have been highlighted as recently as 2024 [35]. These findings are concerning, as low uptake of Oncotype DX testing in breast cancer has implications for chemotherapy receipt and guideline adherence for optimising patient care. One study was identified exploring disparities in utilisation of the Oncotype DX prostate cancer test [31]. As a more recent and less utilised testing modality, this demonstrated similar patterns of disparities in decreased utilisation with increased age; non‐white races and/or ethnicities; lower socioeconomic status; and non‐private insurance.

NGS testing panels were grouped separately for review. Similarly to the Oncotype DX studies, these indicated generally decreased utilisation in non‐white races and/or ethnicities; non‐private insurance status; lower socio‐economic groups; and potentially older age. When included, Asian or Asian/other ethnic groups demonstrated increased utilisation compared to white population comparators in NSCLC studies [43, 46, 47]. This may represent clinician bias in favouring genomic testing where certain socio‐demographic factors have shown evidence of increased likelihood of actionable mutations. Patients of Asian descent with lung cancer have been demonstrated to have a higher burden of EGFR and ALK actionable mutations [57]. Likewise, female gender was associated with increased utilisation in four studies [43, 46, 47, 48]. These studies were all on patients with NSCLC or contained patients with NSCLC. Certain targetable mutations, particularly those in EGFR, are recognised to be more prevalent in female populations [58]. Therefore, this may again demonstrate an element of clinical bias where NGS testing is selective.

In the USA, Medicare national coverage determination for reimbursement for NGS testing commenced in 2018. As such, insufficient time has passed for large numbers of patients benefiting from such to have been included in studies in this review. Reimbursement and the large amount of NGS products now available on markets may aid in utilisation among patients without private insurance in the United States (US) population. In the present review, the cohorts included feature data often from the transition time prior to reimbursement and increased testing product numbers; therefore, the data regarding the impact of these two changes is not yet clear. In many European countries, such NGS panels are reimbursed or free at the point of care, such as in the publicly funded UK National Health Service (NHS). This review lacked data from countries outside of the US, which is both key in our interpretation of the results, with US reimbursement most relevant, and also highlights an area of unmet need in research as data begins to emerge from other healthcare settings.

There was noted to be decreased receipt of testing in patients with lung cancer who were current or former smokers as opposed to never smokers [47]. The reasons for this may be linked to increased smoking rates in lower socio‐economic groups [59] or indeed biases interlinked with smoking causation of lung cancer and attached stigma. Furthermore, it has been recognised that non‐smokers are significantly more likely to demonstrate actionable mutations; therefore, clinicians may again prioritise genomic testing in this cohort [60]. Irrespective of rationale, smoking has been surmised to be a barrier to equitable lung cancer care [61].

In the development of the next generation of precision oncology tools, intrinsic socio‐demographic inequalities must be considered. The initial development of breast cancer Oncotype DX utilised information from cohorts in which only 5%–6% of the patients were Black females [62]. This may in part account for discrepancies in the prognostic value of the test, with decreased accuracy in Black cohorts [63]. Certain novel strategies in cancer research, such as machine learning models and other artificial intelligence tools, are particularly sensitive to exaggerating disparities between groups if trained on stereotyped data with intrinsic bias [64]. Of note, this may be present in supervised machine learning models which are being explored to predict ‘high’ and ‘low’ risk breast cancer Oncotype DX groups without necessitating genomic testing [65, 66]. This further highlights the importance of identifying such inequalities where they occur and the active management of future developments to prevent integration of systemic biases which prevent equity of access to novel cancer advances. As clinician education increases as to the benefits of biomarker testing and precision oncology, it is hoped that equitable access is embedded within the anticipated changes in clinical practice.

This review has many strengths in that it provides a contemporary update since 2018 and the broadening of access to NGS panels for solid tumours on the utilisation of genomic testing by socio‐demographic groups. Testing represents an evolving field in which inequalities have been suspected to exist. Combined, data from over 3,200,000 patients across 7 recent years of publication were considered. However, several limitations do exist both methodological and in terms of the evidence base itself. Firstly, there was heterogeneity of classifications of socio‐demographic factors—such as disparities between ethnicity and race and socio‐economic group measures and terminologies. This created challenges in combining such data meaningfully to draw succinct conclusions. Secondly, studies published between 2018 and 2024 did include data from the mid to late 2000s, predating recent advances in precision oncology. Even when used as comparators, these patients are likely to have had differences in diagnostics and treatment options which may confound results. Furthermore, it is acknowledged that in a number of the presented studies, a mix of earlier genetic testing methodologies such as Sanger sequencing were used. When NGS represented the predominant testing modality as per author agreement these were included. It is recognised that in such older cases, any inequalities of access to testing by socio‐demographic factors represent access to the most modern somatic mutation testing available for those patients at the time. ctDNA testing itself is easier for the patient involved, with a simple blood draw yielding contemporary tumour mutational results. Solid tumour testing requires either an invasive up‐to‐date tumour biopsy or utilisation of older tissue, which is less likely to pass quality testing and yield mutation results or may not demonstrate a complete picture of the mutational landscape of the progressed disease at the time of testing. ORs were used when presented in the studies; however, this was not consistent across studies; therefore, a mix of adjusted and unadjusted ORs, as well as hazard ratios when used by the authors, is present for consideration. A single reviewer completing title and abstract screening may have resulted in human error of study omission; however, this is acceptable by the Cochrane Collaboration [67] and on > 10% review of titles and abstracts author agreement by Kappa coefficient was excellent. Within the National Cancer Database studies [14, 30, 34] included on breast cancer Oncotype DX testing there is likely to have been some overlap of patients included; however, the socio‐demographic factors explored differ sufficiently to have not significantly weighted the synthesis of this review. Certain socio‐economic characteristics (e.g., education, smoking and disability status) were not detailed across many studies and may have added depth to the understanding of existing disparities. Finally, data sets from outside the US, and in a diversity of tumour sites outside breast cancer, were lacking and may give a more comprehensive view of worldwide challenges to equitable testing practices in oncology and the generalisability of this review to a non‐US population.

This review especially highlights the lack of evidence in this field, particularly regarding NGS panels, to comprehensively assess equality of access by socio‐demographic factors. This identifies an important area for future research focus. This should encourage robust utilisation data collection moving forwards internationally in order to mitigate the risk of accentuating disparities in cancer care further. What would also be of particular value are studies in publicly funded healthcare settings where access to care is less dependent on insurance or financial status.

## 5 Conclusions

There are socio‐demographic inequalities in the utilisation of somatic mutation testing in solid tumours. Utilisation is generally lower in patients who are: non‐white; older; with non‐private insurance; and from less affluent socio‐economic groups. Further characterisation and interventions are required to prevent widening of differences in outcome and integration of further systemic inequalities between socio‐demographic groups.

## Author Contributions

**Sarah Rae:** conceptualization (equal), data curation (equal), formal analysis (equal), writing – original draft (lead), writing – review and editing (lead). **Annie Baldwin:** data curation (equal), formal analysis (equal). **Maria Julia Lagonera:** data curation (equal), formal analysis (equal). **Ruth Norris:** conceptualization (equal), data curation (equal), supervision (supporting), writing – review and editing (equal). **Alastair Greystoke:** conceptualization (equal), data curation (equal), formal analysis (equal), supervision (lead), writing – review and editing (equal). **Linda Sharp:** conceptualization (equal), data curation (equal), formal analysis (equal), supervision (lead), writing – review and editing (equal).

## Funding

S.R. receives fellowship funding from the National Institute for Health and Care Research (NIHR). L.S. and R.N. are funded by the NIHR Newcastle Patient Safety Research Collaboration (PSRC). The views expressed are those of the authors and not necessarily those of the NIHR or Department of Health and Social Care.

## Conflicts of Interest

A.G. has received consultancy and speaker fees from Guardant and Foundation Medicine, and is the Clinical Director for Cancer for the North East England and Yorkshire Genomic Medicine Service. The views expressed are those of the authors and not necessarily those of the North East England and Yorkshire Genomic Medicine Service.

## Supporting information


**Data S1:** cam471668‐sup‐0001‐Supinfo.docx.

## Data Availability

All data utilised is available from the authors on reasonable request.

## References

[1] “APPG on Cancer, Cancer Inequalities Report—Macmillan Cancer Support,” (2023), https://www.macmillan.org.uk/documents/getinvolved/campaigns/appg/britainagainstcancer2009/cancerinequalitiesreport.pdf.

[2] Macmillan Cancer Support and NHS Scotland , “Deprivation and Cancer Survival in Scotland: Technical Report,” (2018), https://www.macmillan.org.uk/_images/ISD%20Macmillan%20Deprivation%20Survival%20Technical%20Report_FINAL_tcm9‐308832.pdf.

[3] Macmillan Cancer Support , “Health Inequalities: Time to Talk—Macmillan Cancer Support,” (2023), https://www.macmillan.org.uk/assets/health‐inequalities‐paper‐april‐2019.pdf.

[4] Cancer Research UK , “Deprivation Gradient for Cancer Mortality (2020) Cancer Research UK,” (2023), https://www.cancerresearchuk.org/health‐professional/cancer‐statistics/mortality/deprivation‐gradient.

[5] R. P. Norris , R. Dew , A. Greystoke , A. Todd , and L. Sharp , “Socioeconomic Inequalities in Novel NSCLC Treatments During the Era of Tumor Biomarker‐Guided Therapy: A Population‐Based Cohort Study in a Publicly Funded Health Care System,” Journal of Thoracic Oncology 18, no. 8 (2023): 990–1002, 10.1016/j.jtho.2023.04.018. 37146751

[6] L. Hayes , L. Forrest , J. Adams , et al., “Age‐Related Inequalities in Colon Cancer Treatment Persist Over Time: A Population‐Based Analysis,” Journal of Epidemiology and Community Health 73, no. 1 (2018): 34–41, 10.1136/jech-2018-210842. 30409922

[7] K. Logan , F. Pearson , R. P. Kenny , S. Pandanaboyana , and L. Sharp , “Are Older Patients Less Likely to Be Treated for Pancreatic Cancer? A Systematic Review and Meta‐Analysis,” Cancer Epidemiology 80 (2022): 102215, 10.1016/j.canep.2022.102215. 35901624

[8] R. Lawrenson , S. Seneviratne , N. Scott , T. Peni , C. Brown , and I. Campbell , “Breast Cancer Inequities Between Māori and Non‐Māori Women in Aotearoa/New Zealand,” European Journal of Cancer Care 25, no. 2 (2016): 225–230, 10.1111/ecc.12473. 26918687

[9] J. T. Jørgensen , “The Current Landscape of the FDA Approved Companion Diagnostics,” Translational Oncology 14, no. 6 (2021): 101063, 10.1016/j.tranon.2021.101063. 33714919 PMC7957094

[10] Press Releases , “Guardant Health, Inc.—Guardant Health Secures Coverage From Major US Commercial Health Insurers for Guardant360 Blood Test for Comprehensive Genomic Profiling,” (2024), https://investors.guardanthealth.com/press‐releases/press‐releases/2023/.

[11] Foundation Medicine , “FDA Approves Foundation Medicine's FoundationOneLiquid Cdx, a Comprehensive Pan‐Tumor Liquid Biopsy Test With Multiple Companion Diagnostic Indications for Patients With Advanced Cancer, Foundation Medicine,” (2020), https://www.foundationmedicine.com/press‐releases/fda‐approves‐foundation‐medicine%27s‐foundationone%C2%AEliquid‐cdx,‐a‐comprehensive‐pan‐tumor‐liquid‐biopsy‐test‐with‐multiple‐companion‐diagnostic‐indications‐for‐patients‐with‐advanced‐cancer.

[12] E. A. Klein , M. R. Cooperberg , C. Magi‐Galluzzi , et al., “A 17‐Gene Assay to Predict Prostate Cancer Aggressiveness in the Context of Gleason Grade Heterogeneity, Tumor Multifocality, and Biopsy Undersampling,” European Urology 66, no. 3 (2014): 550–560, 10.1016/j.eururo.2014.05.004. 24836057

[13] “About the Oncotype DX Breast Recurrence Score Test: Oncotype IQ United Kingdom (No Date),” (2024), https://www.oncotypeiq.com/en‐gb/breast‐cancer/healthcare‐professionals/oncotype‐dx‐breast‐recurrence‐score/about‐the‐test?gclid=Cj0KCQiAh8OtBhCQARIsAIkWb69TmEQcXK9NECaOpQul1lCuQlnPi18jCqyhiJvQWadzmlHFRx0COrcaAjAUEALw_wcB.

[14] S. Chen , C. Thacker , S. Wang , K. A. Young , R. L. Hoffman , and J. A. Blansfield , “Adherence Disparities and Utilization Trends of Oncotype DX Assay: A National Cancer Database Study,” Journal of Surgical Research 286 (2023): 65–73, 10.1016/j.jss.2023.01.002. 36758322

[15] R. W. Huey , E. Hawk , and A. C. Offodile , “Mind the Gap: Precision Oncology and Its Potential to Widen Disparities,” Journal of Oncology Practice 15, no. 6 (2019): 301–304. 31112478 10.1200/JOP.19.00102

[16] K. A. McClellan , D. Avard , J. Simard , and B. M. Knoppers , “Personalized Medicine and Access to Health Care: Potential for Inequitable Access?,” European Journal of Human Genetics 21, no. 2 (2013): 143–147. 22781088 10.1038/ejhg.2012.149 PMC3548263

[17] R. P. Norris , R. Dew , L. Sharp , et al., “Are There Socio‐Economic Inequalities in Utilization of Predictive Biomarker Tests and Biological and Precision Therapies for Cancer? A Systematic Review and Meta‐Analysis,” BMC Medicine 18, no. 1 (2020): 282, 10.1186/s12916-020-01753-0. 33092592 PMC7583194

[18] “Company History: Exact Sciences,” (2024), https://www.exactsciences.com/uk/about/company‐history.

[19] J. R. Trosman , S. L. Van Bebber , and K. A. Phillips , “Coverage Policy Development for Personalized Medicine: Private Payer Perspectives on Developing Policy for the 21‐Gene Assay,” Journal of Oncology Practice 6, no. 5 (2010): 238–242, 10.1200/jop.000075. 21197187 PMC2936466

[20] “Our History, Foundation Medicine,” (2024), https://www.foundationmedicine.com/timeline.

[21] D. M. Sheinson , W. B. Wong , C. Flores , S. Ogale , and C. P. Gross , “Association Between Medicare's National Coverage Determination and Utilization of Next‐Generation Sequencing,” JCO Oncology Practice 17, no. 11 (2021): 1023, 10.1200/op.20.01023. PMC8600504 34043456

[22] D. T. Cheng , T. N. Mitchell , and A. Zehir , “Memorial Sloan Kettering‐Integrated Mutation Profiling of Actionable Cancer Targets (MSK‐Impact),” Journal of Molecular Diagnostics 17, no. 3 (2015): 251–264, 10.1016/j.jmoldx.2014.12.006. PMC5808190 25801821

[23] “NHS Genomic Medicine Service. NHS Choices,” (2024), https://www.england.nhs.uk/genomics/nhs‐genomic‐med‐service/.

[24] T. Bhandari , “Medicare Approves Washu Medicine's Whole‐Genome Test for Blood Cancers, Washington University School of Medicine in St. Louis,” (2023), https://medicine.wustl.edu/news/medicare‐approves‐washu‐medicines‐whole‐genome‐test‐for‐blood‐cancers.

[25] Tempus , “Tempus Announces the National Launch of FDA‐Approved xT CDx Test,” (2025), https://www.tempus.com/news/pr/tempus‐announces‐the‐national‐launch‐of‐fda‐approved‐xt‐cdx‐test/#:~:text=%E2%80%9CWe%20are%20thrilled%20to%20broadly,Officer%20of%20Oncology%20at%20Tempus.

[26] Caris Life Sciences , “MI Cancer Seek,” (2024), https://www.carislifesciences.com/physicians/physician‐tests/mi‐cancer‐seek/.

[27] N. Acuna , J. J. Plascak , J. Tsui , A. M. Stroup , and A. A. M. Llanos , “Oncotype DX Test Receipt Among Latina/Hispanic Women With Early Invasive Breast Cancer in New Jersey: A Registry‐Based Study,” International Journal of Environmental Research and Public Health 18, no. 10 (2021): 5116, 10.3390/ijerph18105116. 34065945 PMC8151910

[28] M. R. Dunn , D. Li , M. A. Emerson , et al., “A Latent Class Assessment of Healthcare Access Factors and Disparities in Breast Cancer Care Timeliness,” PLoS Medicine 21, no. 12 (2024): e1004500, 10.1371/journal.pmed.1004500. 39621782 PMC11649116

[29] L. E. Hull , J. A. Lynch , B. B. Berse , et al., “Clinical Impact of 21‐Gene Recurrence Score Test Within the Veterans Health Administration: Utilization and Receipt of Guideline‐Concordant Care,” Clinical Breast Cancer 18, no. 2 (2018): 135–143, 10.1016/j.clbc.2017.11.018. 29306660

[30] K. Iles , M. L. Roberson , P. Spanheimer , et al., “The Impact of Age and Nodal Status on Variations in Oncotype DX Testing and Adjuvant Treatment,” NPJ Breast Cancer 8 (2022): 27, 10.1038/s41523-022-00394-1. 35232996 PMC8888624

[31] N. H. Mukand , E. Chirikova , D. Lichtensztajn , et al., “Assessing Sociodemographic and Regional Disparities in Oncotype DX Genomic Prostate Score Uptake,” Cancer 130, no. 24 (2024): 4298–4305, 10.1002/cncr.35511. 39158464 PMC12688416

[32] K. H. Natsuhara , K. Losk , and T. A. King , “Impact of Genomic Assay Testing and Clinical Factors on Chemotherapy Use After Implementation of Standardized Testing Criteria,” Oncologist 24, no. 5 (2018): 595–602, 10.1634/theoncologist.2018-0154. 30076279 PMC6516114

[33] M. C. Roberts , A. W. Kurian , and V. I. Petkov , “Uptake of the 21‐Gene Assay Among Women With Node‐Positive, Hormone Receptor−Positive Breast Cancer,” Journal of the National Comprehensive Cancer Network 17, no. 6 (2019): 662–668, 10.6004/jnccn.2018.7266. 31200352

[34] N. Y. Ko , M. M. Qureshi , O. T. Oladeru , et al., “Racial Differences in Genomic Testing and Receipt of Endocrine Therapy in Early‐Stage Breast Cancer,” Breast Cancer Research and Treatment 184, no. 3 (2020): 849–859, 10.1007/s10549-020-05888-9. 32888137

[35] S. C. Van Alsten , M. R. Dunn , A. M. Hamilton , et al., “Disparities in OncotypeDx Testing and Subsequent Chemotherapy Receipt by Geography and Socioeconomic Status,” Cancer Epidemiology, Biomarkers & Prevention 33 (2024): 1201, 10.1158/1055-9965.epi-23-1201. PMC11062804 38270534

[36] R. Zipkin , A. Schaefer , and M. Chamberlin , “Surgeon and Medical Oncologist Peer Network Effects on the Uptake of the 21‐Gene Breast Cancer Recurrence Score Assay,” Cancer Medicine 10, no. 4 (2021): 1253–1263, 10.1002/cam4.3720. 33455068 PMC7926024

[37] D. S. Bruno , L. M. Hess , X. Li , E. W. Su , and M. Patel , “Disparities in Biomarker Testing and Clinical Trial Enrollment Among Patients With Lung, Breast, or Colorectal Cancers in the United States,” JCO Precision Oncology 6 (2022): 427, 10.1200/po.21.00427. 35737912

[38] D. S. Bruno , X. Li , and L. M. Hess , “Biomarker Testing, Targeted Therapy and Clinical Trial Participation by Race Among Patients With Lung Cancer: A Real‐World Medicaid Database Study,” JTO Clinical and Research Reports 5, no. 3 (2024): 100643, 10.1016/j.jtocrr.2024.100643. 38496377 PMC10941001

[39] C. Hage Chehade , Y. Jo , G. Gebrael , et al., “Trends and Disparities in Next‐Generation Sequencing in Metastatic Prostate and Urothelial Cancers,” JAMA Network Open 7, no. 7 (2024): e2423186, 10.1001/jamanetworkopen.2024.23186. 39023888 PMC11258596

[40] R. Halder , S. Veeravelli , C. Cheng , R. J. Estrada‐Mendizabal , and A. Recio‐Boiles , “Health Disparities in Presentation, Treatment, Genomic Testing, and Outcomes of Pancreatic Cancer in Hispanic and Non‐Hispanic Patients,” Journal of Racial and Ethnic Health Disparities 10, no. 6 (2023): 3131–3139, 10.1007/s40615-022-01486-1. 37071331 PMC10645638

[41] M. Huang , P. Kamath , M. Schlumbrecht , et al., “Identifying Disparities in Germline and Somatic Testing for Ovarian Cancer,” Gynecologic Oncology 153, no. 2 (2019): 297–303, 10.1016/j.ygyno.2019.03.007. 30890269

[42] S. Peleg Hasson , D. Hershkovitz , L. Adar , et al., Implementation of Comprehensive Genomic Profiling in Ovarian Cancer Patients: A Retrospective Analysis (MDPI, 2022), https://www.mdpi.com/2072‐6694/15/1/218. 10.3390/cancers15010218 PMC9818378 36612212

[43] K. Kehl , C. S. Lathan , B. E. Johnson , et al., “Race, Poverty, and Initial Implementation of Precision Medicine for Lung Cancer,” JNCI Journal of the National Cancer Institute 111, no. 4 (2019): 431–434, 10.1093/jnci/djy202. 30576459 PMC6449167

[44] M. M. M. Khan , M. Khalil , H. Stecko , and T. M. Pawlik , “Disparities in Next‐Generation Genetic Sequencing Among Individuals With Cancer,” Annals of Surgical Oncology 32, no. 2 (2025): 650–652, 10.1245/s10434-024-16464-6. 39523293

[45] S. C. Markt , B. D. Booker , W. Bensken , et al., “Sociodemographic and Clinical Factors Associated With Receipt of Biomarker Testing in Patients With Metastatic Colorectal Cancer,” Cancer Medicine 12, no. 2 (2022): 1850–1859, 10.1002/cam4.4995. 35837788 PMC9883565

[46] C. Meernik , F. Wang , Y. Raveendran , et al., “Association of Race and Ethnicity With Genomic Testing at a Comprehensive Cancer Center in North Carolina,” Cancer Research Communications 4, no. 11 (2024): 2968–2975, 10.1158/2767-9764.CRC-24-0134. 39440958 PMC11570879

[47] C. J. Presley , D. Tang , P. R. Soulos , et al., “Association of Broad‐Based Genomic Sequencing With Survival Among Patients With Advanced Non–Small Cell Lung Cancer in the Community Oncology Setting,” JAMA 320, no. 5 (2018): 469–477, 10.1001/jama.2018.9824. 30088010 PMC6142984

[48] S. Tuminello , W. M. Turner , M. Untalan , T. Ivic‐Pavlicic , R. Flores , and E. Taioli , “Racial and Socioeconomic Disparities in Non‐Small Cell Lung Cancer Molecular Diagnostics Uptake,” Journal of the National Cancer Institute 117, no. 1 (2025): 112–119, 10.1093/jnci/djae225. 39254646 PMC11717419

[49] Y. Zhao , A. Dimou , Z. C. Fogarty , et al., “Real‐World Trends, Rural‐Urban Differences, and Socioeconomic Disparities in Utilization of Narrow Versus Broad Next‐Generation Sequencing Panels,” Cancer Research Communications 4, no. 2 (2024): 303–311, 10.1158/2767-9764.CRC-23-0190. 38276870 PMC10840454

[50] PRISMA , PRISMA Flow Diagram (PRISMA, 2025), https://www.prisma‐statement.org/prisma‐2020‐flow‐diagram.

[51] L. M. Woods , B. Rachet , M. P. Coleman , et al., “Origins of Socio‐Economic Inequalities in Cancer Survival: A Review,” Annals of Oncology 17, no. 1 (2006): 5–19. 16143594 10.1093/annonc/mdj007

[52] M. C. Roberts , M. Weinberger , S. B. Dusetzina , et al., “Racial Variation in Adjuvant Chemotherapy Initiation Among Breast Cancer Patients Receiving Oncotype DX Testing,” Breast Cancer Research and Treatment 153, no. 1 (2015): 191–200, 10.1007/s10549-015-3518-9. 26216535 PMC4562432

[53] B. A. Davis , J. A. Aminawung , M. M. Abu‐Khalaf , et al., “Racial and Ethnic Disparities in Oncotype DX Test Receipt in a Statewide Population‐Based Study,” Journal of the National Comprehensive Cancer Network 15, no. 3 (2017): 346–354, 10.6004/jnccn.2017.0034. 28275035

[54] J. Jasem , A. Amini , and R. Rabinovitch , “21‐Gene Recurrence Score Assay as a Predictor of Adjuvant Chemotherapy Administration for Early‐Stage Breast Cancer: An Analysis of Use, Therapeutic Implications, and Disparity Profile,” Journal of Clinical Oncology 34, no. 17 (2016): 1995–2002, 10.1200/jco.2015.65.0887. 27001563 PMC4966515

[55] C. Chen , R. Dhanda , W.‐Y. Tseng , M. Forsyth , and D. A. Patt , “Evaluating Use Characteristics for the Oncotype DX 21‐Gene Recurrence Score and Concordance With Chemotherapy Use in Early‐Stage Breast Cancer,” Journal of Oncology Practice 9, no. 4 (2013): 182–187, 10.1200/jop.2012.000638. 23942918 PMC3710166

[56] M. C. Roberts and S. B. Dusetzina , “Use and Costs for Tumour Gene Expression Profiling Panels in the Management of Breast Cancer From 2006 to 2012: Implications for Genomic Test Adoption Among Private Payers,” Journal of Oncology Practice 11, no. 4 (2015): 273–277, 10.1200/jop.2015.003624. 26105668

[57] S. Li , Y. Choi , Z. Gong , et al., “Comprehensive Characterization of Oncogenic Drivers in Aian Lung Adenocarcinoma,” Journal of Thoracic Oncology 11, no. 12 (2016): 2129–2140, 10.1016/j.jtho.2016.08.142. 27615396

[58] R. Rosell , T. Moran , C. Queralt , et al., “Screening for Epidermal Growth Factor Receptor Mutations in Lung Cancer,” New England Journal of Medicine 361, no. 10 (2009): 958–967, 10.1056/NEJMoa0904554. 19692684

[59] S. C. Hitchman , G. T. Fong , M. P. Zanna , J. F. Thrasher , J. Chung‐Hall , and M. Siahpush , “‘Socioeconomic Status and Smokers’ Number of Smoking Friends: Findings From the International Tobacco Control (ITC) Four Country Survey,” Drug and Alcohol Dependence 143 (2014): 158–166, 10.1016/j.drugalcdep.2014.07.019. 25156228 PMC4209373

[60] S. Devarakonda , Y. Li , R. F. Martins , et al., “Genomic Profiling of Lung Adenocarcinoma in Never‐Smokers,” Journal of Clinical Oncology 39, no. 33 (2021): 3747–3758, 10.1200/JCO.21.01691. 34591593 PMC8601276

[61] J. Dunn , G. Garvey , P. C. Valery , et al., “Barriers to Lung Cancer Care: Health Professionals Perspectives,” Supportive Care in Cancer 25, no. 2 (2016): 497–504, 10.1007/s00520-016-3428-3. 27726030 PMC5196009

[62] J. DePolo , “Lower Oncotype DX Scores Seem Less Accurate for Black Women With Hormone Receptor‐Positive Breast Cancer, Breastcancer.org—Breast Cancer Information and Support,” (2023), https://www.breastcancer.org/research‐news/black‐women‐oncotype‐less‐accurate.

[63] K. F. Hoskins , O. C. Danciu , and N. Y. Ko , “Association of Race/Ethnicity and the 21‐Gene Recurrence Score With Breast Cancer–Specific Mortality Among US Women,” JAMA Oncology 7, no. 3 (2021): 370–378, 10.1001/jamaoncol.2020.7320. 33475714 PMC7821091

[64] “Measuring Stereotype Harm From Machine Learning Errors Requires,” (2023), https://eaamo.org/papers/EAAMO23_paper_19.pdf.

[65] K. R. Pawloski , M. Gonen , H. Y. Wen , et al., “Supervised Machine Learning Model to Predict Oncotype DX Risk Category in Patients Over Age 50,” Breast Cancer Research and Treatment 191, no. 2 (2021): 423–430, 10.1007/s10549-021-06443-w. 34751852 PMC9281430

[66] V. Romeo , R. Cuocolo , and L. Sanduzzi , “MRI Radiomics and Machine Learning for the Prediction of Oncotype DX Recurrence Score in Invasive Breast Cancer,” Cancers 15, no. 6 (2023): 1840, 10.3390/cancers15061840. 36980724 PMC10047199

[67] T., H.J.P , Cochrane Handbook for Systematic Reviews of Interventions (Wiley‐Blackwell, 2020).

