# Supplementary material for: Are There Socio‐Demographic Inequalities in the Utilisation of Tumour and ctDNA Somatic Mutation Testing in Solid Tumours? A Systematic Review
Source: Cancer Med. 2026 Mar 13;15(3):e71668. doi: 10.1002/cam4.71668 (PMC13093295; doi:10.1002/cam4.71668)
Supplement: Supplementary file 1 — Data S1: cam471668‐sup‐0001‐Supinfo.docx. [file CAM4-15-e71668-s001.docx]

**SUPPLEMENT**

**ARE THERE SOCIO-DEMOGRAPHIC INEQUALITIES IN THE UTILISATION OF TUMOUR AND ctDNA SOMATIC MUTATION TESTING IN SOLID TUMOURS? A SYSTEMATIC REVIEW**

Sarah Rae MBChB BSc^1^, Annie Baldwin MBChB MRes^2^, Mariajulia Lagonera MD BSc ^2^, Ruth Norris PhD ^3^, Alastair Greystoke MBChB PhD^1,2^, Linda Sharp PhD^3^

1. Newcastle University, Newcastle upon Tyne NE1 7RU, UK

2. Sir Bobby Robson Cancer Trials Research Centre, Northern Centre for Cancer Care, Freeman Hospital, Freeman Road, Newcastle upon Tyne NE7 7DN, UK

3. Population Health Sciences Institute, Newcastle University, Newcastle University, Newcastle upon Tyne NE1 7RU, UK

**Supplementary Methods**

**Supplementary Table S1: PRISMA Checklist**

**
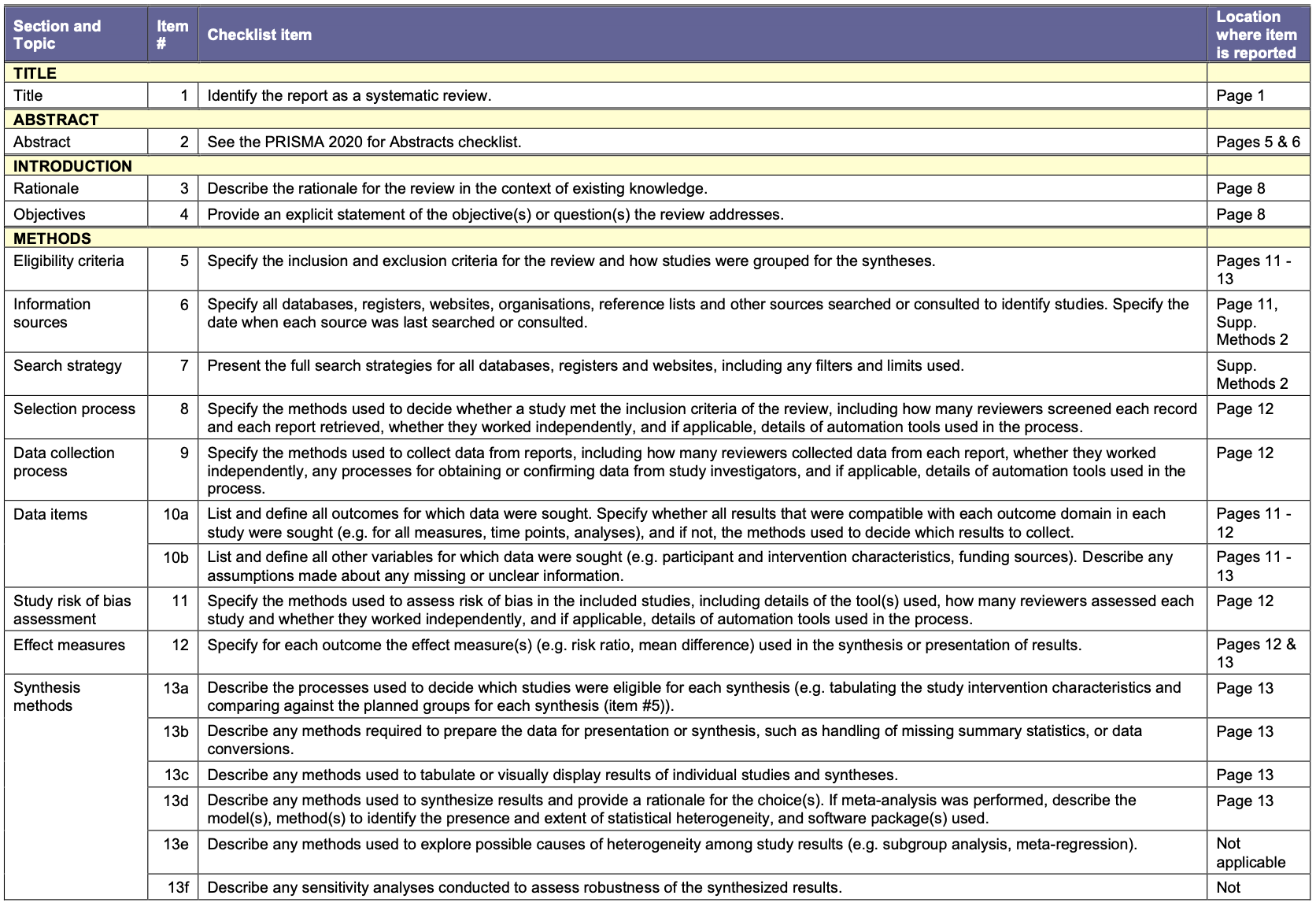
**

**
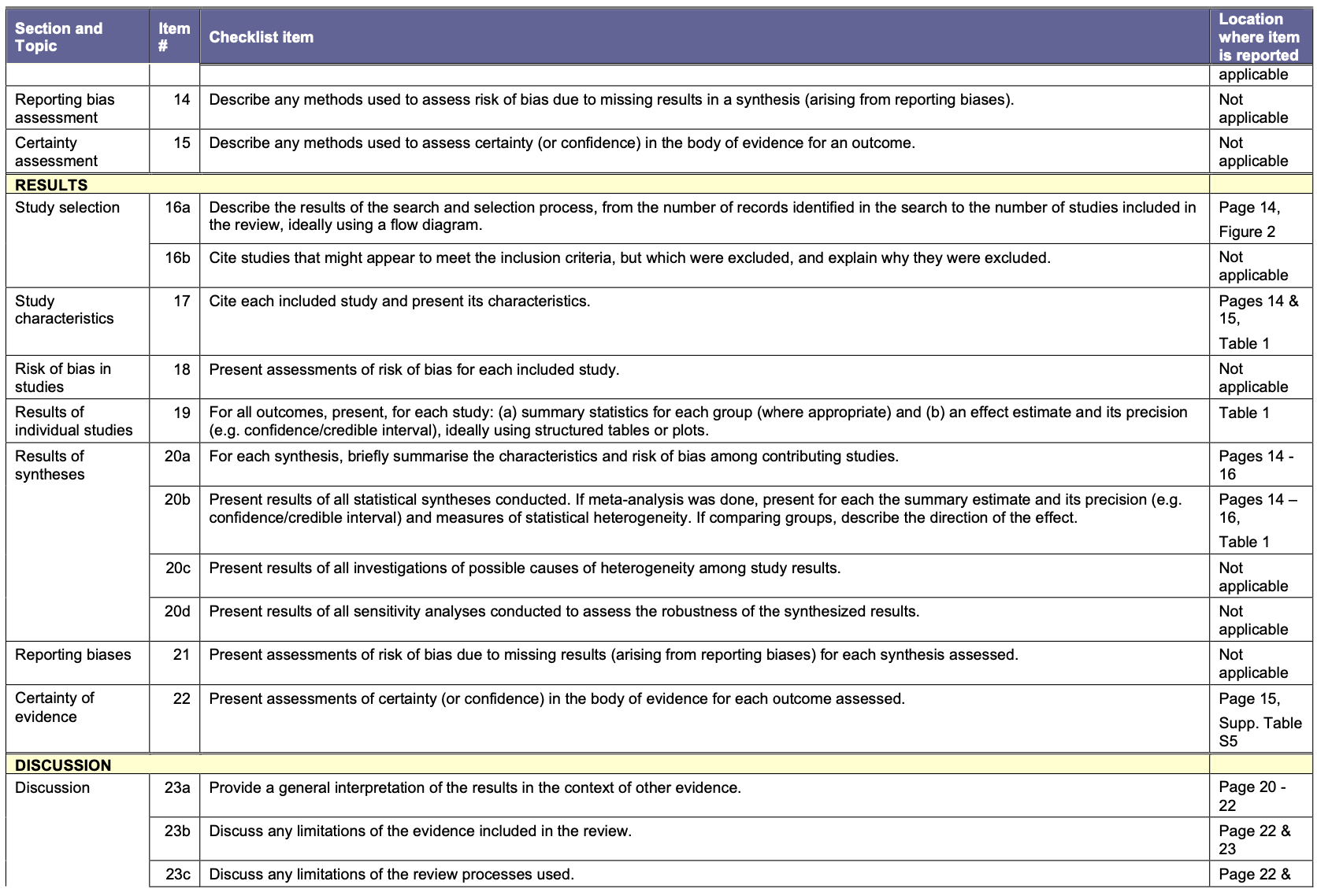

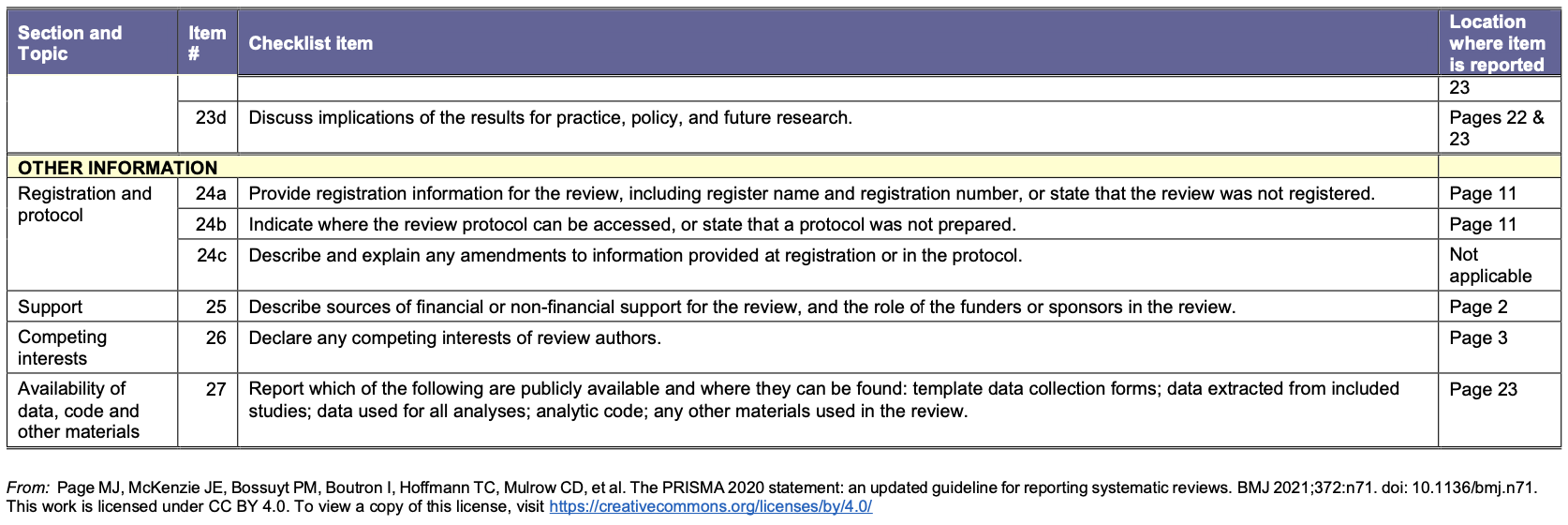
**

**Supplementary Table S2**: Database Search Strategies

**A. MEDLINE search strategy 05/12/2023, updated to 31/03/2025**

| **Search Term** | **Number Retrieved** |
| --- | --- |
| 1. Treatment Barrier.mp 2. health services accessibility / or healthcare disparities/ 3. Non-receipt.mp 4. Non-initation.mp 5. Treatment Pattern.mp 6. Practice Patterns, Physicians’/ 7. Utilisation Pattern.mp 8. Utilization Pattern.mp 9. Uptake.mp 10. Provision.mp 11. Receipt of.mp 12. Treatment with.mp. 13. Receive.mp. 14. Non-use.mp. 15. Treatment Receipt.mp. 16. Receipt.mp 17. Initiate.mp. 18. Treatment Utilisation.mp. 19. Treatment Utilization.mp. 20. Utilization.mp. 21. “Procedures and Techniques Utilization”/ 22. Utilisation.mp. 23. Access.mp. 24. Underutilisation.mp. 25. Underutilization.mp. 26. Socioeconomic Status.mp. 27. Socioeconomic factors/ or economic factors/ or economic status/ or poverty/ or social class/ or low socioeconomic status 28. Poverty Areas.mp. 29. poverty areas/ 30. Social Class.mp. 31. Social Class/ 32. Social Mobility.mp. 33. Social Mobility/ 34. Index of Multiple Deprivation.mp. 35. Socioeconomic Position.mp. 36. Carstairs Index.mp. 37. Townsend Index.mp 38. Area Level Deprivation.mp. 39. Population groups/ or residence characteristics/ or catchment area, health/ or home environment/ or housing / or independent living/ or neighbourhood characteristics/ 40. Inequalities.mp. 41. Healthcare Disparities/ 42. Deprivation.mp. 43. Poverty Level.mp. 44. Income Level.mp. 45. Income/ 46. Household Income.mp. 47. Education Status.mp. 48. education status/ or academic failure/ or literacy/ 49. Education Level.mp. 50. Employment Status.mp. 51. Employment/ 52. Employment Characteristic.mp. 53. Environmental biomarker.mp 54. Environmental Biomrkers/ 55. Unemployment.mp. 56. Unemployment/ 57. Race ethnicity.mp. 58. Population groups/ or African people/ or Asian people/ or black people/ or Caribbean people/ or central American people/ or European people/ or “middle eastern and north African”/ or north American people/ or “American indian or Alaska native”/ or population groups, us/ or ethnicity/ or “Hispanic or latino”/ or racial groups/ or oceanians/ or south American people/ or white people/ 59. Race.mp. 60. Smoker.mp. 61. Smokers/ 62. Non-smoker.mp. 63. Non-smokers/ 64. Smoking History.mp. 65. Gender.mp. 66. Gender Identity/ 67. Male/ 68. Female/ 69. Age.mp. 70. Age Factors/ 71. Health Insurance Status.mp. 72. insurance coverage/ or universal health insurance/ 73. Socioeconomic.mp. 74. Medicare.mp. 75. medicaid/ or medicare/ 76. Insurance Coverage.mp. 77. insurance coverage/ or universal health insurance 78. Health Plan Type.mp. 79. Public Insurance.mp. 80. Medically Uninsured.mp. 81. Medically Uninsured/ 82. Commercial Insurance.mp. 83. Private Insurance.mp. 84. Military Insurance.mp. 85. Other Insurance.mp. 86. Income Category.mp. 87. Median Income.mp. 88. Education Demographic.mp. 89. Patient Education as Topic/ 90. Education Variable.mp. 91. education status/ or academic failure/ or literacy/ 92. Disabled Variable.mp. 93. Personalised Medicine.mp. 94. Precision Medicine/ 95. Personalized medicine.mp. 96. Tailored Medicine.mp. 97. Biomarkers, Tumor/ 98. Genomic Medicine.mp. 99. Genomic Medicine/ 100. Genetic Testing/ 101. Precision Cancer Care.mp. 102. Stratified Medicine.mp. 103. Precision Oncology.mp. 104. Precision Medicine.mp. 105. Precision Medicine/ 106. Genomic Testing.mp. 107. Genomics/ 108. Targeted Test.mp 109. Biomarker Test.mp. 110. Molecular Test.mp. 111. Mutation Test.mp. 112. Test Trend.mp. 113. Test Pattern.mp. 114. Genetic Profile.mp. 115. genetic profile/ 116. ctDNA.mp. 117. Circulating Tumor DNA/ 118. ctDNA.mp. 119. Cell-Free Nucleic Acids/ 120. Foundation Medicine.mp. 121. Genomic panel.mp. 122. Next-generation sequencing.mp. 123. High-Throughput Nucleotide Sequencing/ 124. Comprehensive Genomic Profiling.mp. 125. Genomic Profiling.mp 126. liquid biopsy.mp 127. Liquid Biopsy 128. Guardant.mp. 129. MSK Impact.mp. 130. MSK Access.mp. 131. Companion diagnositic.mp 132. Predictive biomarker.mp. 133. Caris.mp. 134. whole exome sequencing.mp. 135. whole genome sequencing/ or exome sequencing/ 136. targeted sequencing.mp. 137. single gene tests.mp. 138. (1-25) OR 139. (26-92) OR 140. (93 – 137) OR 141. 138 AND 139 AND 140 142. Neoplasms/ 143. Cancer*.mp 144. ‘neoplas*.mp. 145. ‘oncolo*.mp. 146. Cancer research.mp. 147. Tumour.mp. 148. Tumours.mp. 149. Malignancy.mp. 150. Malignancies.mp 151. Solid tumours.mp. 152. Medical Oncology/ 153. Medical oncology.mp. 154. Clinical oncology.mp. 155. Precision oncology.mp. 156. Cancer genetics.mp 157. (142-156) OR 158. 141 AND 157 159. Limit 158 to yr=”2018-March2025” | 75  103008  151  135  804  67214  68  601  454944  98354  25764  610258  278560  3431  303  25781  81308  71  1076  280863  823  27862  439807  291  2737  56847  241926  6873  6697  50581  45034  1789  1134  929  3873  49  105  291  81514  30109  22469  102281  2312  4061  35096  13772  1189  62244  20963  11170  50887  2  29  789  11341  7835  28556  266455  144219  14276  4554  2589  716  11746  431407  22371  9390447  9663392  9996501  472418  1185  19917  259718  77314  68160  22948  19917  57  2124  8229  8155  1498  5870  51  332  280  1207  30  88362  28  62244  0  1942  27001  18834  112  178802  2040  99  45314  32  385  2371  41340  27001  1412  67273  39  318  1389  855  44  427  3210  302  4426  2681  3959  3541  156  25  58284  46594  813  3268  8481  2776  44  193  4  756  5868  103  18836  17444  3409  57  2258560  15295201  12480  451341  507859  2306241  3408473  231377  19790  242939  138373  181107  149789  7441  24113  28009  7355  2371  2099  4245027  8070  3353 |

**B. CINAHL search strategy 05/12/2023, updated to 31/03/2025**

| **Search Term** | **Number Retrieved** |
| --- | --- |
| 1. “Treatment Barrier” 2. “Non-receipt” 3. “Non-initiation” 4. “Treatment Pattern” 5. “Utilisation Pattern” 6. “Utilization Pattern” 7. “Uptake” 8. “Provision” 9. “Receipt of” 10. “Treatment with” 11. “Receive” 12. “Non-use” 13. “Treatment Receipt” 14. “Receipt” 15. “Initiate” 16. “Treatment Utilisation” 17. “Treatment Utilization” 18. “Utilization” 19. “Utilisation” 20. “Access” 21. “Underutilisation” 22. “Underutilization” 23. “Socioeconomic Status” 24. “Socioeconomic Factors 25. “Poverty” 26. “Poverty Areas” 27. “Social Class” 28. “Social Mobility” 29. “Index of Multiple Deprivation” 30. “Socioeconomic Position: 31. “Carstairs Index” 32. “Townsend Index” 33. “Area Level Deprivation” 34. “Inequalities” 35. “Deprivation” 36. “Poverty Level” 37. “Income Level” 38. “Household Income” 39. “Education Status” 40. “Education Level” 41. “Employment Status” 42. “Employment Characteristic” 43. “Environmental biomarker” 44. “Unemployed” 45. “Race Ethnicity” 46. “Smoker” 47. “Non-smoker” 48. “Smoking History” 49. “Gender” 50. “Male” 51. “Female” 52. “Age” 53. “Health Insurance Status” 54. “Socioeconomic” 55. “Medicare” 56. “Medicaid” 57. “Insurance Coverage” 58. “Health Plan Type” 59. “Public Insurance” 60. “Medically Uninsured” 61. “Commercial Insurance” 62. “Private Insurance” 63. “Military Insurance” 64. “Other Insurance” 65. “Income Category” 66. “Median Income” 67. “Education Demographic” 68. “Education Variable” 69. “Disabled Variable” 70. “Personalised Medicine” 71. “Personalized Medicine” 72. “Tailored Medicine” 73. “Genomic Medicine” 74. “Precision Cancer Care” 75. “Stratified Medicine” 76. “Precision Oncology” 77. “Precision Medicine” 78. “Genetic Testing” 79. “Genomic Testing” 80. “Targeted Test” 81. “Biomarker Test” 82. “Molecular Test” 83. “Mutation Test” 84. “Test Trend” 85. “Test Pattern” 86. “Genetic Profile” 87. “ctDNA” 88. “circulating tumour DNA” 89. “cfDNA” 90. “cell free DNA” 91. “Foundation Medicine” 92. “Genomic panel” 93. “Next-generation sequencing” 94. “Comprehensive Genomic Profiling” 95. “Genomic Profiling” 96. “liquid biopsy” 97. “Guardant” 98. “MSK Impact” 99. “MSK Access” 100. “companion diagnostic” 101. “predictive biomarker” 102. “Caris” 103. “whole exome sequencing” 104. “Whole genome sequencing” 105. “targeted sequencing” 106. “single gene tests” 107. “cancer” 108. “neoplas*” 109. “oncolog*” 110. “cancer research” 111. “tumour” 112. “tumours” 113. “malignancy” 114. “malignancies” 115. “solid tumour” 116. “medical oncology” 117. “clinical oncology” 118. “precision oncology” 119. “cancer genetics” 120. (1-22) OR 121. (23-69) OR 122. (70-106) OR 123. (107-119) OR 124. 120 AND 121 AND 122 AND 123 | 24  72  63  174  17  160  53351  54896  12,629  1320950  99292  1408  154  12629  13579  40  623  272529  10219  188597  143  1234  40966  106725  39847  3725  16021  707  422  2010  22  42  149  14676  19065  11570  1715  11353  5073  13361  25494  3135  21  5159  15407  4492  636  3477  161946  1991675  2287403  1774350  630  131154  67600  47544  17247  26  1144  7405  5090  2893  21  168  121  564  21  11  238  352  23017  21  465  14  88  492  7219  14073  497  9  118  215  132  6  40  818  982  1686  851  1778  35  1  6544  266  743  1575  18  35  2  185  1215  108  2601  2127  453  11  558249  567462  103763  4778  98197  85138  28571  23439  1529  8034  5283  492  500  1808576  3243375  56964  808378  2793 |

**C. PsychINFO search strategy 06/12/2023, updated to 31/03/2025**

| **Search Term** | **Number Retrieved** |
| --- | --- |
| 1. Treatment Barrier.mp. 2. health services accessibility / or healthcare disparities/ 3. Non-receipt.mp. 4. Non-initiation.mp. 5. Treatment Pattern.mp. 6. Practice Patterns, Physicians’/ 7. Utilisation Pattern.mp. 8. Utilization Pattern.mp. 9. Uptake.mp. 10. Provision.mp. 11. Receipt of.mp. 12. Treatment with.mp. 13. Receive.mp. 14. Non-use.mp. 15. Treatment Receipt.mp. 16. Receipt.mp. 17. Initiate.mp. 18. Treatment Utilisation.mp. 19. Treatment Utilization.mp. 20. Utilization.mp. 21. “Procedures and Techniques Utilization”/ 22. Utilisation.mp. 23. Access.mp. 24. Underutilisation.mp. 25. Underutilization.mp. 26. Socioeconomic Status.mp. 27. Socioeconomic factors/ or economic factors/ or economic status/ or poverty/ or social class/ or low socioeconomic status 28. Poverty Areas.mp. 29. poverty areas/ 30. Social Class.mp. 31. Social Class/ 32. Social Mobility.mp. 33. Social Mobility/ 34. Index of Multiple Deprivation.mp. 35. Socioeconomic Position.mp. 36. Carstairs Index.mp. 37. Townsend Index.mp. 38. Area Level deprivation.mp. 39. population groups/ or residence characteristics/ or catchment areas, health/ or home environment/ or housing/ or independent living/ or neighbourhood characteristics/ 40. Inequalities.mp. 41. Healthcare Disparities/ 42. Deprivation.mp. 43. Poverty Level.mp. 44. Income Level.mp. 45. Income/ 46. Household Income.mp 47. Education Status.mp. 48. educational status/ or academic failure/ or literacy/ 49. Education Level.mp. 50. Employment Status.mp. 51. Employment/ 52. Employment Characteristic.mp. 53. Environmental biomarker.mp. 54. Environmental Biomarkers/ 55. Unemployed.mp. 56. Unemployment/ 57. Race Ethnicity.mp 58. population groups/ or African people/ or Asian people/ or black people/ or Caribbean people/ or central American people/ or European people/ or “middle eastern and north Africans”/ or north American people/ or “American Indian or Alaska native”/ or population groups, us/ or ethnicity 59. Race.mp. 60. Smoker.mp. 61. Smokers/ 62. Non-smoker.mp. 63. Non-Smokers/ 64. Smoking History.mp. 65. Gender.mp. 66. Gender Identity/ 67. Male/ 68. Female/ 69. Age.mp. 70. Age Factors/ 71. Health Insurance Status.mp. 72. insurance coverage/ or universal health insurance/ 73. Socioeconomic.mp. 74. Medicare.mp. 75. medicaid/ or medicare/ 76. Insurance Coverage.mp. 77. Insurance coverage/ or universal health insurance/ 78. Health Plan Type.mp. 79. Public Insurance.mp. 80. Medically Uninsured.mp. 81. Medically Uninsured/ 82. Commercial Insurance.mp. 83. Private Insurance.mp. 84. Military Insurance.mp. 85. Other Insurance.mp. 86. Income Category.mp. 87. Median Income.mp. 88. Education Demographic.mp. 89. Patient Education as Topic/ 90. Education Variable.mp. 91. educational status/ or academic failure/ or literacy/ 92. Disabled Variable.mp. 93. Personalised Medicine.mp. 94. Precision Medicine/ 95. Personalized Medicine.mp. 96. Tailored Medicine.mp. 97. Biomarkers, Tumor. 98. Genomic Medicine.mp. 99. Genomic Medicine/ 100. Genetic Medicine/ 101. Precision Cancer Care.mp. 102. Stratified Medicine.mp. 103. Precision Oncology.mp. 104. Precision Medicine.mp. 105. Precision Medicine/ 106. Genomic Testing.mp. 107. Genomics/ 108. Targeted Test.mp. 109. Biomarker Test.mp. 110. Molecular Test.mp. 111. Mutation Test.mp. 112. Test Trend.mp. 113. Test Pattern.mp. 114. Genetic Profile.mp. 115. Genetic profile/ 116. ctDNA.mp. 117. Circulating Tumor DNA/ 118. cfDNA.mp. 119. Cell-Free Nucleic Acids/ 120. Foundation Medicine.mp. 121. Genomic panel.mp. 122. Next-generation sequencing.mp. 123. High-Throughput Nucleotide Sequencing/ 124. Comprehensive Genomic Profiling.mp 125. Genomic Profiling.mp. 126. liquid biopsy.mp. 127. Liquid Biopsy/ 128. Guardant.mp. 129. MSK Impact.mp. 130. MSK Access.mp. 131. companion diagnostic.mp. 132. predictive biomarkers.mp. 133. Caris.mp. 134. whole exome sequencing.mp. 135. whole genome sequencing/ or exome sequencing/ 136. targeted sequencing.mp. 137. single gene tests.mp 138. (1-25) OR 139. (26-92) OR 140. (93-137) OR 141. 138 AND 139 AND 140 142. Neoplasms/ 143. cancer*.mp. 144. ‘neoplas*.mp. 145. ‘oncolog*.mp. 146. cancer research.mp. 147. tumour.mp. 148. tumours.mp. 149. malignancy.mp. 150. malignancies.mp 151. solid tumours.mp. 152. Medical Oncology/ 153. Medical oncology.mp. 154. clinical oncology.mp. 155. precision oncology.mp. 156. cancer genetics.mp. 157. (142-156) OR 158. 141 AND 157 159. Limit 158 to yr=”2018-March2025” | 56  0  37  35  50  0  7  61  31593  45333  8749  45300  65680  1096  136  9169  13950  28  950  53325  0  3610  135918  64  1211  53207  21245  1836  757  23773  8054  3189  1920  368  1382  13  24  83  23148  14370  0  38212  894  21365  0  4992  499  19793  8895  23172  19030  2  0  0  8034  5140  15028  19408  87716  3041  0  294  0  1339  270289  11198  0  0  681355  0  348  0  102020  6593  5057  3618  0  13  486  689  468  171  1015  8  58  70  309  47  0  23  19793  0  111  736  1189  3  0  151  0  2219  0  37  12  1893  736  149  1209  15  20  8  6  1  222  204  0  2  0  21  0  1  1  857  0  3  40  28  0  0  0  0  6  125  9  1012  0  91  4  369469  1098624  8591  195  44502  76042  68732  14905  695  1934  1016  1498  1177  38  0  1065  181  12  203  92851  62  28 |

**D. Supplementary Search Strategy:** Example Scopus 05/12/2023, updated to 31/03/2025

“Treatment Barrier” OR “Non-receipt” OR “Non-initiation” OR “Treatment Pattern” OR “Utilisation Pattern” OR “Utilization Pattern” OR “Uptake” OR “Provision” OR “Receipt of” OR “Treatment with” OR “Receive” OR “Non-use” OR “Treatment Receipt” OR “Receipt” OR “Initiate” OR “Treatment Utilisation” OR “Treatment Utilization” OR “Utilization” OR “Utilisation” OR “Access” OR “Underutilisation” OR “Underutilization”

**AND**

“Socioeconomic Status” OR “Socioeconomic Factors” OR “Poverty” OR “Poverty Areas” OR “Social Class” OR “Social Mobility” OR “Index of Multiple Deprivation” OR “Socioeconomic Position” OR “Carstairs Index” OR “Townsend Index” OR “Area Level Deprivation” OR “Inequalities” OR “Deprivation” OR “Poverty Level” OR “Income Level” OR “Household Income” OR “Education Status” OR “Education Level” OR “Employment Status” OR “Employment Characteristic” OR “Environmental biomarker” OR “Unemployed” OR “Race Ethnicity” OR “Smoker” OR “Non-smoker” OR “Smoking History” OR “Gender” OR “Male” OR “Female” OR “Age” OR “Health Insurance Status” OR “Socioeconomic” OR “Medicare” OR “Medicaid” OR “Insurance Coverage” OR “Health Plan Type” OR “Public Insurance” OR “Medically Uninsured” OR “Commercial Insurance” OR “Private Insurance” OR “Military Insurance” OR “Other Insurance” OR “Income Category” OR “Median Income” OR “Education Demographic” OR “Education Variable” OR “Disabled Variable”

**AND**

“Personalised Medicine” OR “Personalized Medicine” OR “Tailored Medicine” OR “Genomic Medicine” OR “Precision Cancer Care” OR “Stratified Medicine” OR “Precision Oncology” OR “Precision Medicine” OR “Genetic Testing” OR “Genomic Testing” OR “Targeted Test” OR “Biomarker Test” OR “Molecular Test” OR “Mutation Test” OR “Test Trend” OR “Test Pattern” OR “Genetic Profile” OR “ctDNA” OR “cfDNA” OR “Foundation Medicine” OR “Genomic panel” OR “Next-generation sequencing” OR “Comprehensive Genomic Profiling” OR “Genom(ic) Profiling” OR “liquid biopsy” OR “Guardant” OR “MSK Impact” OR “MSK Access” OR “companion diagnostic” OR “predictive biomarker” OR “Caris” OR “whole exome sequencing” OR “Whole genome sequencing” OR “targeted sequencing” OR “single gene tests”

**AND**

“cancer*” OR “neoplas*” OR “oncolog*” OR “cancer research” OR “tumour” OR “tumours” OR “malignancy” OR “malignancies” OR “solid tumours” OR “medical oncology” OR “clinical oncology” OR “precision oncology” OR “cancer genetics”

**
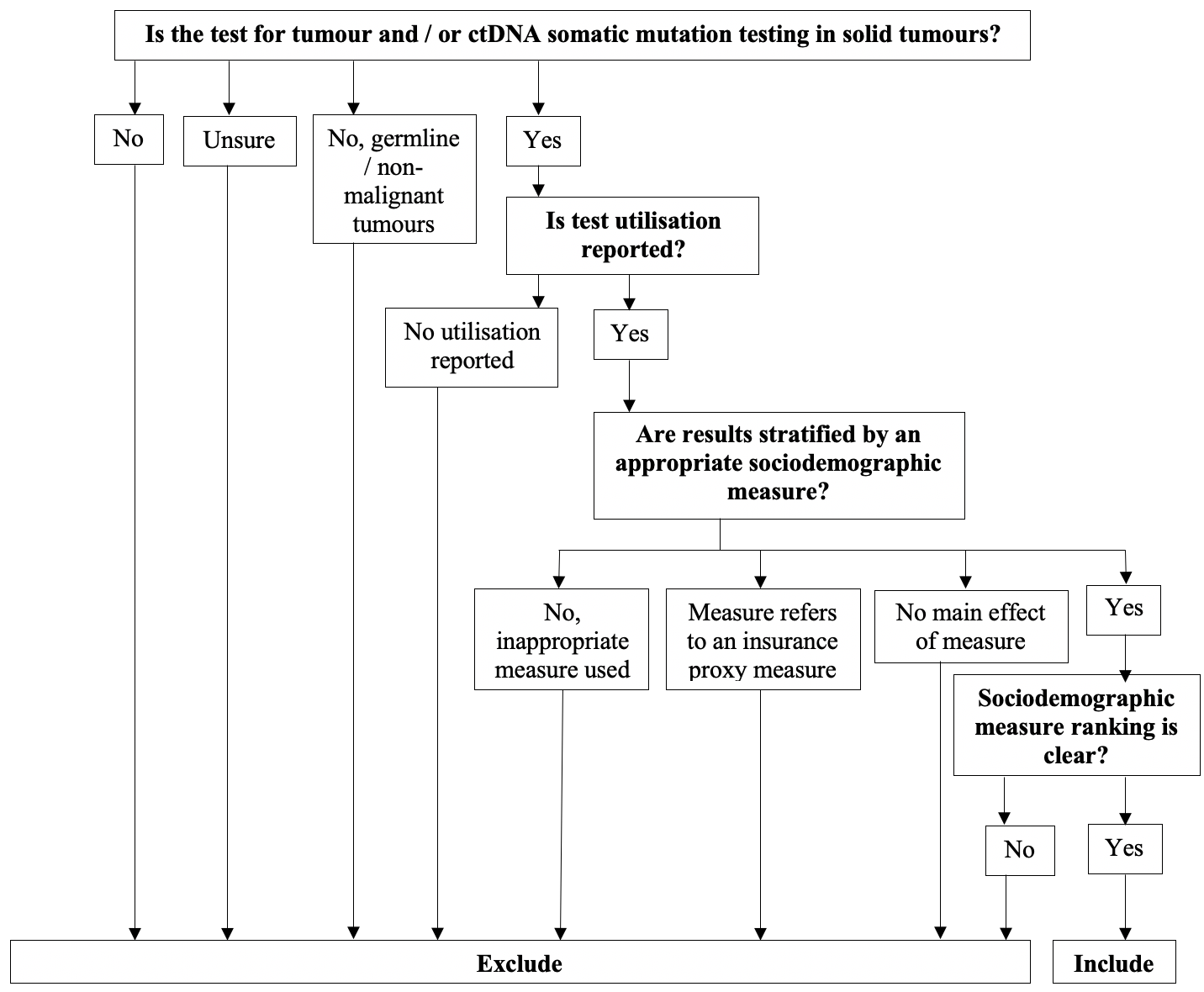
**

**Supplementary Figure S3:** Full text study inclusion / exclusion criteria decision tree

**Supplementary Table S4:** Quality Appraisal Tool


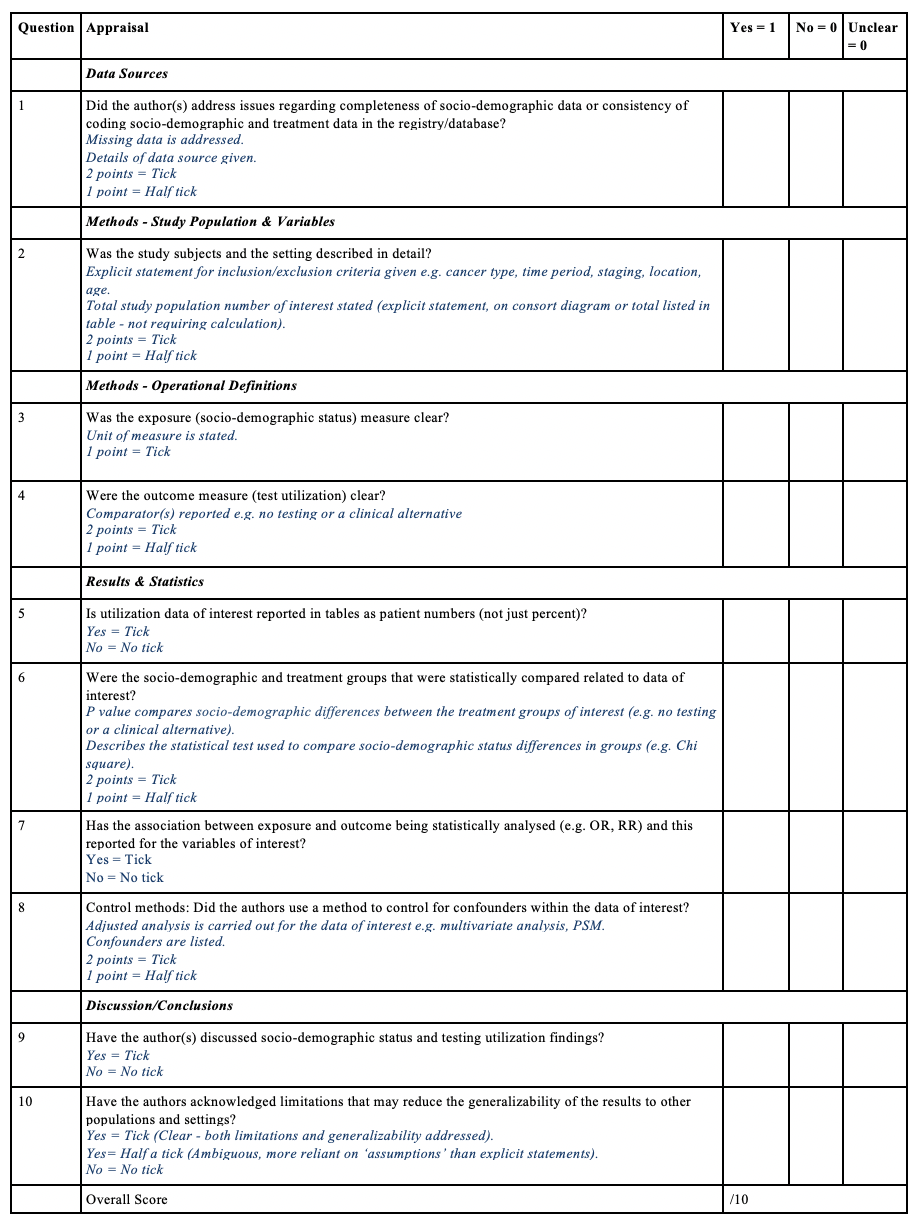


**Supplementary Table S5**: Study quality appraisal results for the 24 included studies

| **Test Type** | **Study** | **Q1** | **Q2** | **Q3** | **Q4** | **Q5** | **Q6** | **Q7** | **Q8** | **Q9** | **Q10** | **Total** |
| --- | --- | --- | --- | --- | --- | --- | --- | --- | --- | --- | --- | --- |
| **Oncotype DX: Breast Cancer** | Acuna *et al* (2021) [26]  Chen *et al* (2022) [12]  Dunn *et al* (2024) [X]  Hull *et al* (2018) [27]  Iles et al (2022) [28]  Mukand *et al* (2024) [X]  Natsuhara *et al* (2019) [29]  Roberts *et al* (2019) [30]  Ko *et al* (2020) [31]  Van Alsten *et al* (2024) [X]  Zipkin et al (2020) [32] | 0.5  0.5  0.5  0.5  0.5  1  0.5  1  1  0.5  0.5 | 1  1  1  1  1  1  1  1  1  1  1 | 1  1  1  1  1  1  1  1  1  1  1 | 1  1  1  1  1  1  1  0.5  1  1  1 | 1  1  1  1  1  1  1  0  1  1  1 | 1  1  0  0.5  0.5  1  1  0  1  0.5  1 | 1  1  0.5  1  1  1  1  1  1  0  0 | 1  0.5  0  0  0  1  0.5  0.5  0.5  0  0 | 1  1  1  1  1  1  1  1  1  1  1 | 1  1  1  1  0.5  0.5  1  1  1  1  1 | **9.5**  **9**  **7**  **8**  **7.5**  **9.5**  **9**  **7**  **9.5**  **7**  **7.5** |
| **NGS Testing: All tumour types** | Bruno *et al* (2022) [33]  Bruno et al (2024) [x]  Chehade *et al* (2024) [X]  Halder *et al* (2022) [34]  Huang *et al* (2019) [35]  Hasson *et al* (2022) [36]  Kehl *et al* (2019) [37]  Khan et al (2024) [X]  Markt *et al* (2022) [38]  Meernik *et al (*2024) [X]  Presley *et al* (2018) [39]  Tuminello *et al* (2024) [X]  Zhao *et al* (2024) [X] | 0.5  1  0.5  1  1  1  0.5  0.5  1  1  1  1  1 | 1  1  1  1  1  0.5  1  1  1  1  1  1  1 | 1  1  1  1  1  1  1  1  1  1  1  1  1 | 1  1  1  1  1  1  1  1  1  1  1  1  1 | 1  1  0  1  1  1  0  1  1  1  1  1  1 | 1  0.5  1  1  1  1  1  1  1  1  1  1  1 | 0  0  1  0  1  0  1  1  1  1  0  1  1 | 0  0  0  0  1  0  0.5  1  0  0.5  0  1  1 | 1  1  1  1  1  0  1  1  1  1  1  1  1 | 1  0.5  0.5  1  1  1  0.5  0.5  1  1  1  0.5  0.5 | **7.5**  **7**  **7**  **8**  **10**  **6.5**  **7.5**  **9**  **9**  **9.5**  **8**  **9.5**  **9.5** |
